# CDH1 Orchestrates Anabolic Events to Promote Cell Cycle Initiation

**DOI:** 10.1002/advs.202507584

**Published:** 2025-09-29

**Authors:** Yun‐Zi Mao, Jiao‐Jiao Zhang, Meng‐Qi You, Han Wang, Yan Lin, Yuan Fang, Hui‐Lu Zhang, Yuan‐Yuan Qu, Qian Zhou, Meng Wang, Gang Li, Zi‐Fan Guo, Meng Xu, Jie Liu, Peng‐Cheng Lin, Yi‐Yuan Yuan, Yu Kang, Shi‐Min Zhao, Wei Xu

**Affiliations:** ^1^ The Obstetrics and Gynecology Hospital of Fudan University Shanghai Key Lab of Reproduction and Development Shanghai Key Lab of Female Reproductive Endocrine Related Diseases, Shanghai Key Laboratory of Metabolic Remodeling and Health State Key Laboratory of Genetics and Development of Complex Phenotypes, and Institutes of Biomedical Sciences, Fudan Unviersity Shanghai 200433 China; ^2^ Shanghai Institute of Endocrine and Metabolic Diseases Ruijin Hospital Shanghai Jiao Tong University School of Medicine Shanghai 200025 China; ^3^ Department of Liver Surgery Zhongshan Hospital of Fudan University Shanghai 200032 China; ^4^ Department of Digestive Diseases Huashan Hospital of Fudan University Shanghai 200040 China; ^5^ Department of Urology Fudan University Shanghai Cancer Center, Fudan University Shanghai 200032 China; ^6^ Key Laboratory for Tibet Plateau Phytochemistry of Qinghai Province, College of Pharmacy Qinghai University for Nationalities Xining 810007 China

**Keywords:** anabolism, CDH1, cell cycle, R5P

## Abstract

Cell proliferation requires anabolic supports. How the cell cycle integrates anabolism remains poorly understood. Herein, it is identified that G1‐phase regulator cell division cycle 20‐like protein 1 (CDH1) coordinates anabolic events to ensure cell cycle initiation. CDH1 degrades Von Hippel–Lindau (VHL), concomitantly activates hypoxia‐inducible factor 1α (HIF1*α*), which enhances angiogenesis and glucose metabolism, and activates mitochondrial lactyltransferase alanyl tRNA synthetase (AARS2), which lactylates and inactivates pyruvate dehydrogenase E1 subunit alpha 1 (PDHA1), thereby conserving anabolites. Among the CDH1‐accumulated anabolites, ribose‐5‐phosphate (R5P) binds to transketolase‐like‐1 (TKTL1) to bridge CDH1 to cyclin‐dependent kinase 2 (CDK2) and Skp1‐Cullin‐F‐box and *β*‐transducin repeat‐containing protein (SCF*^β^* ^‐TRCP^) complex, thereby facilitating CDH1 phosphorylation and degradation to promote cell cycle initiation. This CDH1‐VHL‐HIF1*α*/AARS2‐R5P/TKTL1 circuit is supported by the observation that low R5P levels and high CDH1 expression correlate with proliferating cancer cells and tissues. Moreover, it is demonstrated that an artificial R5P signal, generated by ribose‐5‐sulfate (R5S), sensitizes cancer cells to apoptosis by initiating the cell cycle in the absence of sufficient anabolite supply. These suggest that cancer signatures, including the Warburg effect and angiogenesis, are intrinsically driven by CDH1.

## Graphical abstract

Cell proliferation requires the integration of cell cycle and anabolic signals. G1‐phase regulator CDH1 coordinates anabolism through the CDH1‐VHL‐AARS2/HIF1*α* axis, while CDH1‐accumulated R5P is sensed by TKTL1 to facilitate CDH1 degradation and cell cycle initiation, constituting a CDH1‐centered regulatory circuit. Pseudo R5P signal generated by R5S promotes cell cycle and sensitizes cancer to chemotherapy.

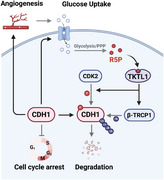

## 1 Introduction

Stimulated angiogenesis and enhanced glucose uptake are key metabolic reprogramming events in cancer cells.^[^ ^1^ ^]^ Additionally, a high glycolytic rate and partially inhibited oxidative phosphorylation (OXPHOS), collectively known as the Warburg effect,^[^ ^2^, ^3^ ^]^ are widely recognized as hallmarks of cancer metabolism. The significance of these coordinated anabolic metabolic reprogramming events is underscored by the observation that inhibition of angiogenesis,^[^ ^4^ ^]^ glucose uptake,^[^ ^5^ ^]^ or the Warburg effect can exert anti‐cancer effects.^[^ ^3^ ^]^ However, the precise drivers of these anabolic adaptations remain a subject of ongoing debate.

HIF1*α*, which transcriptionally induces vascular endothelial growth factor (VEGF) to promote angiogenesis,^[^ ^6^ ^]^ upregulates glucose transporter 1 (GLUT1),^[^ ^7^ ^]^ and upregulates glycolytic and pentose phosphate pathway (PPP) enzymes, is currently considered as a central driver of cancer metabolic reprogramming. This model is particularly relevant in solid tumors, where hypoxic environments prevail. However, non‐hypoxic cancer cells, such as those cultured under normoxic conditions,^[^ ^8^ ^]^ small early‐stage solid tumors,^[^ ^9^ ^]^ and non‐solid tumors like leukemia,^[^ ^10^ ^]^ also exhibit the Warburg effect. These findings suggest the existence of alternative regulators, beyond HIF1*α*, that drive these metabolic reprogramming events. Intriguingly, proliferating non‐cancerous cells, including embryonic, stem, and activated T cells,^[^ ^11^ ^]^ also exhibit enhanced angiogenesis, glucose uptake, and the Warburg effect. These observations imply that the Warburg effect may serve as a general proliferative signature, raising the possibility that cell cycle‐regulating factors could intrinsically drive these metabolic adaptations.

Anabolites, particularly intermediates from glycolysis and PPP, are elevated during the G1 phase of the cell cycle.^[^ ^12^ ^]^ During this phase, cells express CDH1,^[^ ^13^ ^]^ preparing them for adequate anabolite supply to support cell cycle initiation. CDH1 accomplishes this by forming the anaphase‐promoting complex/cyclosome‐CDH1 complex (APC/C^CDH1^), which targets and degrades regulators such as Tome‐1, Skp2, and Ets2 for degradtion, thereby leading to the accumulation of Wee1,^[^ ^14^ ^]^ p21, and p27,^[^ ^15^ ^]^ and decreasing cyclin D1 expression.^[^ ^16^ ^]^ Once the prerequisites of cell proliferation, including anabolites sufficiency, are met, CDH1 is phosphorylated by E‐type cyclins‐activated CDK2. This makes CDH1 a preferred substrate of either the APC/C^CDH1^ complex for self‐degradation, or SCF*^β^* ^‐TRCP^ E3 ligase complex for degradation,^[^ ^17^ ^]^ ultimately facilitating progression through the G1/S checkpoint and the initiation of a new cell cycle.

Despite extensive knowledge, two major questions remain unanswered regarding how cells coordinate anabolite sufficiency during the G1 phase. First, beyond increasing anabolite production, do mechanisms that preserve anabolites, such as inhibiting OXPHOS to decrease anabolite consumption, contribute significantly to anabolite accumulation? Second, how do cells sense anabolite sufficiency and integrate this signal into the decision to initiate the cell cycle? Our previous work has suggested that AARS2 inhibits OXPHOS and promotes cardiomyocyte proliferation,^[^ ^18^, ^19^ ^]^ indicating that OXPHOS inhibition may play a role in cell cycle initiation. Additionally, since DNA replication, the first major anabolic process in a new cell cycle, begins with *de novo* nucleotide synthesis in the late G1 phase,^[^ ^20^ ^]^ anabolites required for nucleotide synthesis may signal anabolite sufficiency.^[^ ^21^ ^]^ Notably, R5P, the precursor for *de novo* nucleotide synthesis, is regulated by the G1‐phase regulator CDH1 through the degradation of TKTL1,^[^ ^22^ ^]^ further supporting the hypothesis that CDH1 regulates anabolite accumulation and sensing during cell cycle initiation.

In the current study, we demonstrate that CDH1 plays a central role in the accumulation and sensing of anabolites required for cell cycle initiation.

## 2 Results

### 2.1 De Novo Nucleotide Synthesis Depletes R5P in Cancer Tissue and Proliferating Cells

**Figure 1:**
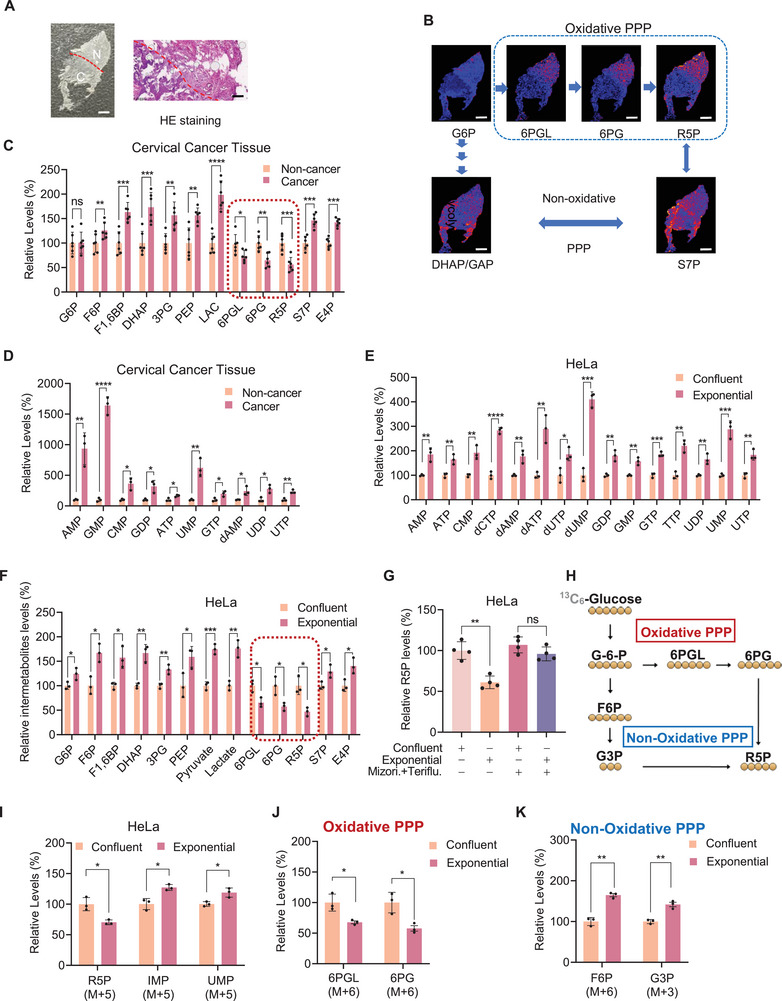
*De novo* nucleotide synthesis depletes R5P in cancer tissue and proliferating cells. A,B) Mass spectrometry imaging (MSI) revealed decreased oxidative PPP metabolites in cervical cancer tissue. Optical microscopy image (left) of cervical cancer tissue and its adjacent non‐cancer tissue were histologically confirmed using hematoxylin and eosin (H&E) staining (A). Scale bar, 2 mm (left), 500 µm (right); oxidative PPP metabolites (B) in both cancer and adjacent non‐cancer tissues were detected using MSI. Scale bar, 2 mm. The quantification of the images was shown in Figure S1B (Supporting Information). C–F) Targeted metabolites assay revealed decreased oxidative PPP metabolites and increased nucleotides in cancer tissue and proliferating cells. The levels of oxidative PPP metabolites, non‐oxidative PPP, nucleotides, and glycolytic metabolites were compared using targeted LC‐MS between cancer and adjacent non‐cancer tissues (C, *n* = 6; D, *n* = 3) and between exponential growing and confluent grown HeLa cells (E,F; *n* = 3). G6P: Glucose‐6‐phosphate, F6P: fructose‐6‐phosphate, F1,6BP: fructose 1,6‐bisphosphate, DHAP: dihydroxyacetone phosphate, 3PG: 3‐phosphoglycerate, PEP: phosphoenolpyruvate, LAC: lactate, 6PGL: 6‐phosphogluconolactone, 6PG: 6‐phosphogluconate, S7P: sedoheptulose 7‐phosphate, R5P: ribose 5‐phosphate, E4P: erythrose 4‐Phosphate. G) Nucleotide synthesis blockade prevented the decrease in oxidative PPP metabolites in proliferating HeLa cells. R5P levels were assayed in exponentially and confluently grown HeLa cells under the absence and presence of mizoribine and teriflunomide to inhibit nucleotide synthesis simultaneously (*n* = 4). H) Schematic of the conversion of ^13^C‐glucose to ^13^C‐R5P and intermediates through oxidative and non‐oxidative PPP. I,J) Isotope‐based metabolic flux analysis revealed increased nucleotide synthesis and a preference for non‐oxidative pentose phosphate pathway flux in proliferating cells. The levels of ^13^C‐labeled R5P, IMP, UMP (I), oxidative PPP metabolites (J), and non‐oxidative PPP metabolites (K) were compared using LC‐MS between exponential growing and confluent grown HeLa cells (*n* = 3). All data are presented as mean ± S.E.M. Statistical significance was assessed using two‐tailed paired (C,D) and unpaired Student's *t*‐test (E,F,G,I,J,K). *p < 0.05, **p < 0.01, ***p < 0.001, ****p < 0.0001; ns, not significant.

To investigate the metabolic changes in cervical cancer, we employed mass spectrometry imaging (MSI) to visualize in vivo metabolites in both cancerous and adjacent non‐cancerous cervical tissues (**Figure** **1**A, Table S1, Supporting Information). Fatty acids, including FA18:1, FA20:4, PI38:4, and PE38:4, were found to be significantly elevated in cancer tissues (Figure S1A, Supporting Information). These findings align with the accumulation of fatty acids in cancers and validate the reliability of the MSI analysis for metabolic profiling.^[^ ^23^ ^]^ Contrary to the prevailing notion that proliferating cells accumulate anabolites, our MSI analysis revealed lower levels of R5P and its upstream oxidative branch PPP intermediates, 6‐phosphogluconolactone (6PGL) and 6‐phosphogluconate (6PG), in cancer tissues compared to adjacent non‐cancerous tissues. In contrast, neighboring metabolites, including those from the non‐oxidative branch of the PPP (sedoheptulose‐7‐phosphate [S7P]) and glycolytic intermediates (glucose 6‐phosphate [G6P] and dihydroxyacetone/glyceraldehyde‐3‐phosphate [DHAP/GAP]), were increased in cancer tissues compared to adjacent non‐cancerous tissues (Figure 1B; Figure S1B, Supporting Information).

To corroborate this observation, we conducted targeted metabolomic assays to quantify glycolytic and PPP metabolites in cancer tissues and their corresponding adjacent non‐cancerous tissues from six patients diagnosed with cervical cancer (Table S2, Supporting Information). Consistent with the MSI results, we observed that 6PGL, 6PG, and R5P levels were significantly reduced, while most other glycolytic and PPP metabolites were elevated in cancer tissues compared to non‐cancerous tissues (Figure 1C). These suggest that either the oxidative branch of the PPP is inhibited, or R5P is depleted, or both, in proliferating cervical cancer cells. Further analysis revealed that the levels of nucleotides, which are synthesized *de novo* using R5P and are upregulated during cell proliferation, were higher in cancer tissues than in adjacent non‐cancerous tissues (Figure 1D). This suggests that the consumption of R5P for nucleotide synthesis may account for the reduced levels of 6PGL, 6PG, and R5P observed in cancer tissues.

In exponentially proliferating human cervical cancer HeLa cells, SiHa cells, and cervical intraepithelial neoplasia Ect1/E6E7 cells, we observed lower R5P levels accompained by higher nucleotide levels and nucleotide upstream anabolite levels (Figure 1E,F; Figure S1C,D, Supporting Information). This was accompanied by a reduction in 6PGL, 6PG, and R5P levels compared to confluent cells (Figure 1F), which serve as a model for non‐proliferating cells (Figure S1E, Supporting Information). Exponentially proliferating HeLa cells had increased glucose‐6‐phosphate dehydrogenase (G6PD) activity, transketolase (TKT) activity, and NADPH levels (Figure S1F–H, Supporting Information). Furthermore, simultaneous inhibition of *de novo* pyrimidine and purine synthesis using teriflunomide and mizoribine (Figure S1I,J, Supporting Information)^[^ ^24^, ^25^ ^]^ which reduced proliferation rates (Figure S1K, Supporting Information), resulted in a significantly smaller decrease in R5P levels in exponentially growing HeLa cells (Figure 1G).

To further investigate the reprogramming of R5P metabolism, we employed isotope‐labeled glucose tracing (Figure 1H). Exponentially proliferating HeLa cells exhibited reduced levels of labeled oxidative PPP intermediates (6PGL and 6PG) as well as R5P, while displaying elevated levels of labeled non‐oxidative PPP intermediates (F6P and G3P), IMP, and UMP (Figure 1I–K). Collectively, these findings suggest the non‐oxidative PPP pathway functions to support *de novo* nucleotide biosynthesis during the rapid proliferation of cancer cells.

### 2.2 There Exists a TKTL1‐CDH1‐VHL‐R5P Regulating Circuit

**Figure 2:**
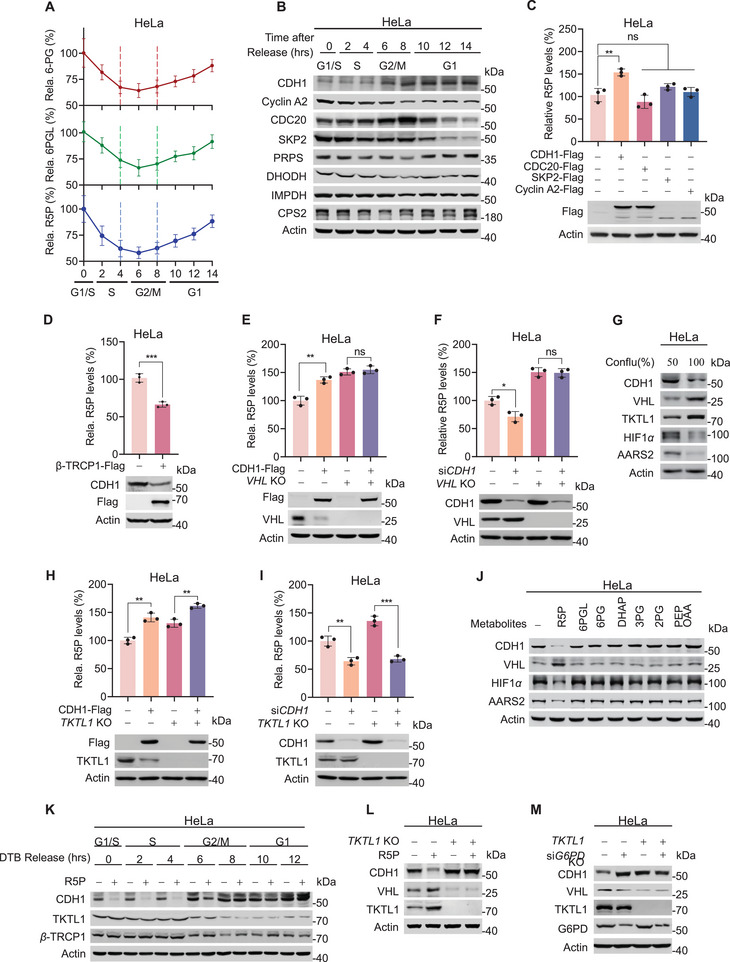
CDH1 upregulates R5P. A–D) CDH1 increases and *β*‐TRCP1 decreases R5P levels. DTB synchronized G1/S HeLa cells were released (time 0), and the levels of metabolites (A, *n* = 3) and cell cycle‐regulating proteins (B) were determined at different times after release. Times that correspond to cell cycle phases were confirmed using flow cytometry (see Figure S2A, Supporting Information). R5P levels were determined using LC‐MS in HeLa cells expressing CDH1, CDC20, SKP2, cyclin A2 (C, *n* = 3), or *β*‐TRCP1 (D, *n* = 3). E,F) CDH1 regulates R5P in a VHL‐dependent manner. R5P levels in HeLa and *VHL* KO HeLa cells were detected with either CDH1 overexpression (E, *n* = 3) or *CDH1* knockdown (F, *n* = 3). G) High CDH1 levels in proliferating cells. Levels of CDH1, VHL, and TKTL1 were detected in exponentially and confluently grown HeLa cells. H,I) TKTL1 is dispensable for CDH1 to regulate R5P levels. R5P levels were detected using LC‐MS and compared with HeLa and *TKTL1* KO HeLa cells under both CDH1 overexpression (H, *n* = 3) and *CDH1* silencing (I, *n* = 3). J) R5P specifically decreases CDH1 levels. Effects of metabolites, namely R5P, 6PGL, 6PG, DHAP, 3PG, 2PG, PEP, and OAA, on CDH1 levels were detected in HeLa cells. K) R5P decreases inversely correlated to CDH1 levels. DTB‐synchronized G1/S HeLa cells were released by DTB removal (time 0). Levels of CDH1 and TKTL1 were determined at different times after DTB removal, with or without R5P treatment. L,M) R5P decreases CDH1 levels in a TKTL1‐dependent manner. Levels of CDH1 were detected in HeLa cells and *TKTL1* KO HeLa cells without or with R5P treatment (L) and *G6PD*‐silencing (M), respectively. All data are presented as mean ± S.E.M. Statistical significance was assessed using two‐way ANOVA (C,E,F,H,I) and two‐tailed unpaired Student's *t‐*test (D). *p < 0.05, **p < 0.01, ***p < 0.001; ns, not significant.

Proliferation‐dependent fluctuations in the levels of R5P, 6PGL, and 6PG prompted us to monitor their dynamics during cell cycle progression. HeLa cells were synchronized at the G1/S boundary using a double thymidine block (DTB) (Figure S2A, Supporting Information), followed by release into the cell cycle. The levels of R5P, 6PGL, and 6PG decreased during the late G1 and S phases (**Figure** **2**A), further supporting the notion that *de novo* nucleotide synthesis, which begins in the late G1 phase and persists through early G2 phase,^[^ ^20^, ^26^, ^27^ ^]^ depletes R5P. Among the G1‐phase regulators, CDH1 and its substrates (Cyclin A2, CDC20, and SKP2) exhibited phase‐dependent variations (Figure 2B), with only CDH1 increasing cellular R5P when ectopically expressed in HeLa, SiHa, and Ect1/E6E7 cells (Figure 2C; Figure S2B,C, Supporting Information). Moreover, overexpressing SCF*^β^* ^‐TRCP^, the E3 ligase of CDH1, led to a reduction in both CDH1 and R5P levels (Figure 2D). These findings support the hypothesis that CDH1 plays a role in upregulating R5P. However, the ability of CDH1 overexpression to increase R5P (Figure 2E) and *CDH1* knockdown to decrease R5P (Figure 2F) was abrogated by the depletion of its substrate, *VHL* in HeLa cells,^[^ ^28^ ^]^ suggesting that CDH1 regulates R5P via VHL.

Additionally, APC/C^CDH1^ is known to degrade TKTL1 to modulate R5P levels.^[^ ^29^ ^]^ While both VHL and TKTL1 were downregulated in proliferating HeLa cells and SiHa cells, but not significantly in Ect1/E6E7 cells (Figure 2G; Figure S2D,E, Supporting Information), deletion of *TKTL1* resulted in elevated R5P levels, without affecting CDH1's ability to upregulate R5P (Figure 2H,I). This indicates that TKTL1 acts independently, or upstream of CDH1 to regulate R5P. Notably, only R5P, but not 6PGL, 6PG, or other tested glycolytic metabolites was capable of decreasing CDH1 and inducing changes in the levels of CDH1, VHL, HIF1*α*, and AARS2, a VHL target (Figure 2J).^[^ ^18^ ^]^ Supplementation with R5P into HeLa cell culture media to increase intracellular R5P levels threefold (Figure S2F, Supporting Information) led to a reduction in CDH1 levels in G1/S, S, and early G2/M phases, during which TKTL1 levels were high. While it had negligible effects on CDH1 in late G2/M and early G1 phases, when TKTL1 levels were low (Figure 2K). Furthermore, the regulatory effects of R5P on CDH1 were abolished in *TKTL1*‐null cells (Figure 2L,M), suggesting that R5P regulates CDH1 expression via TKTL1.

To confirm the role of the TKTL1‐CDH1‐VHL axis in R5P‐mediated nucleotide synthesis, we performed ^13^C_6_‐glucose labeling experiments to track M+5 labeling in R5P and nucleotides following *TKTL1* knockout, *CDH1* knockdown, and *VHL* knockout (Figure S2G, Supporting Information). Reduced M+5 labeling R5P and increased M+5 labeling nucleotides were observed during rapid cell proliferation (Figure 1I), which was eliminated by *TKTL1* knockout, *CDH1* knockdown, or *VHL* knockout (Figure S2H–J, Supporting Information). Taken together, these results support the existence of a TKTL1‐CDH1‐VHL‐R5P regulatory circuit.

### 2.3 R5P Promotes CDH1 Ubiquitination

**Figure 3:**
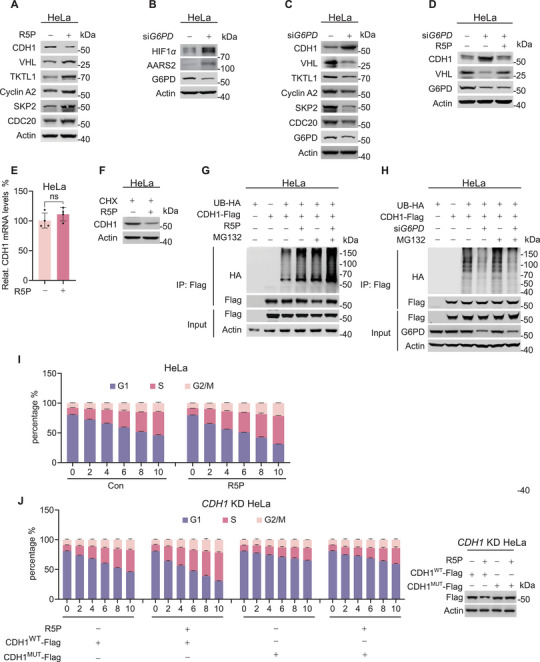
R5P induces CDH1 ubiquitination. A) R5P decreases CDH1 levels. The cellular protein levels of CDH1 and its substrates VHL, TKTL1, Cyclin A2, SKP2, and CDC20 were measured in HeLa cells with or without R5P treatment. B) R5P regulates cellular HIF1*α* and AARS2 levels. Levels of HIF1*α* and AARS2 were detected in HeLa cells with or without *G6PD* silencing with siRNA. C) *G6PD* silencing increases CDH1 levels. Cellular protein levels of CDH1 and its substrates were detected in HeLa and *G6PD*‐silencing HeLa cells. D) R5P rescues *G6PD* silencing‐induced CDH1 and ‐suppressed VHL. CDH1 and VHL protein levels were measured in HeLa and *G6PD*‐silencing HeLa cells in the absence or presence of R5P. E) R5P does not change CDH1 transcription. CDH1 mRNA levels were measured in HeLa cells, with and without R5P treatment (*n* = 4). F) R5P regulates CDH1 through mechanisms other than translation. CDH1 levels in HeLa cells cultured with or without R5P were determined after blocking protein synthesis using cycloheximide (CHX). G,H) R5P regulates CDH1 ubiquitination. Ubiquitination levels of CDH1 were determined in R5P‐treated (G) or *G6PD*‐silenced HeLa cells (H) in the absence and presence of MG132. All ubiquitination levels were compared with those in HeLa cells ectopically expressing only CDH1. I) R5P speeds up the G1/S transition. The numbers of cells in the G1, S, and G2/M phases of HeLa cells were detected under the absence and presence of R5P in the media (*n* = 3). J) R5P speeds up the G1/S transition dependent of CDH1 degradation. The number of cells in the G1, S, and G2/M phases of HeLa cells expressing CDH1^WT^ and non‐degradable CDH1^MUT^ were detected under the absence and presence of R5P in the media (*n* = 3). All data are presented as mean ± S.E.M. Statistical significance was assessed using a two‐tailed unpaired Student's *t*‐test (E). ns, not significant.

Supplementation with R5P resulted in a decrease in CDH1 levels and consistently increased the levels of CDH1 substrates, including VHL (**Figure** **3**A; Figure S3A, Supporting Information).^[^ ^27^ ^]^ The ability of R5P to increase VHL levels was further confirmed by the downregulation of HIF1*α* and AARS2, known VHL substrates that regulate metabolism (Figure 2J), following R5P supplementation. In contrast, knockdown of *G6PD*, which reduced cellular R5P by more than 25% (Figure S3B,C, Supporting Information), led to the upregulation of HIF1*α* and AARS2 (Figure 3B). Conversely, *G6PD* knockdown increased CDH1 and decreased CDH1 substrate levels (Figure 3C). Furthermore, the reduction in VHL levels induced by *G6PD* knockdown was reversed upon R5P supplementation (Figure 3D), confirming that R5P modulates CDH1 and its substrates.

R5P supplementation did not alter CDH1 transcription in HeLa cells (Figure 3E). Additionally, inhibition of protein translation using cycloheximide (CHX) failed to prevent R5P from decreasing CDH1 levels in HeLa cell lysates (Figure 3F). However, the proteasome inhibitor MG132 enhanced the ubiquitination of ectopically expressed CDH1, as R5P did (Figure 3G). Conversely, *G6PD* knockdown, which depletes R5P, led to a decrease in CDH1 ubiquitination (Figure 3H). Consistent with these findings, the ubiquitination of the CDH1 substrate VHL was decreased upon R5P supplementation and increased following *G6PD* knockdown (Figure S3D,E, Supporting Information). Taken together, these results support the hypothesis that R5P downregulates CDH1 by promoting its ubiquitination.

Consistent with *CDH1* knockdown promoting cell cycle progression (Figure S3F, Supporting Information), R5P treatment facilitated the transition from G1 to S phase, thereby accelerating cell cycle progression in serum‐deprived, synchronized HeLa cells (Figure 3I). Furthermore, in *CDH1*‐knockdown cell lines, putting back a degradation‐resistant mutant not only blocks the transition from G1 to S phase but also renders cell cycle progression insensitive to R5P treatment as the control cells (Figure 3J).^[^ ^28^ ^]^ Collectively, these supported that CDH1 ubiquitination is promoted by R5P.

### 2.4 TKTL1 Binds R5P to Promote SCF*^β^* ^‐TRCP^‐Mediated CDH1 Degradation

**Figure 4:**
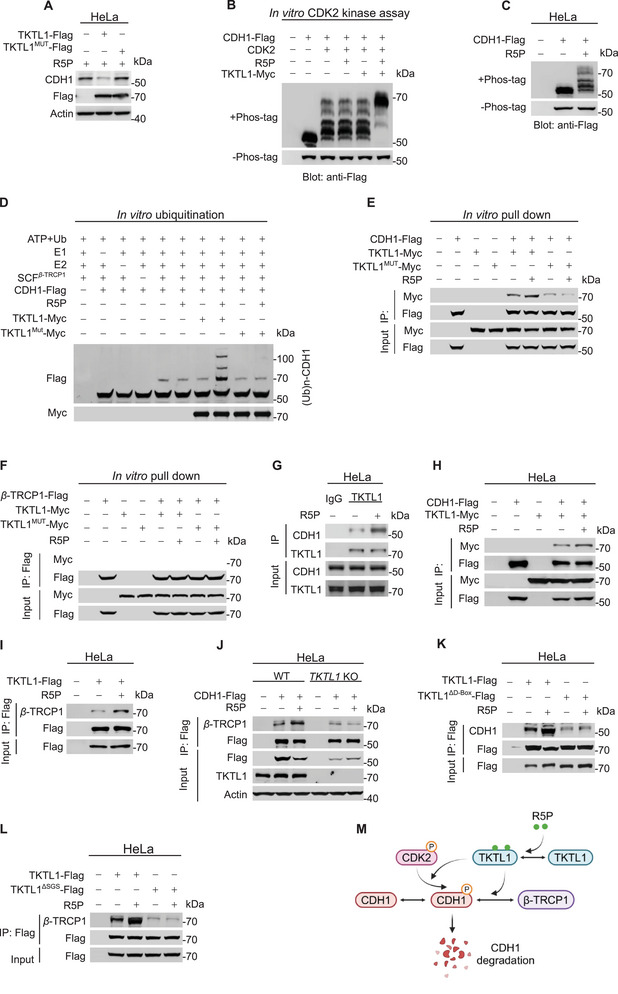
R5P binds to TKTL1 to promote CDH1 ubiquitination by SCF*^β^* ^‐TRCP1^. A) R5P binding is required for TKTL1 to decrease CDH1. Levels of CDH1 were compared between HeLa cells, HeLa cells overexpressing TKTL1, and HeLa cells overexpressing R5P‐binding defective TKTL1 (TKTL1^MUT^). To enhance its effects, R5P was added to the culture medium. B) CDH1 phosphorylation by CDK2 is enhanced by the presence of both TKTL1 and R5P. Phosphorylation of recombinant CDH1 by recombinant CDK2 was tested in vitro in the presence of recombinant TKTL1, R5P, or both. The phosphorylation of CDH1 was detected using Phos‐tag SDS‐PAGE. C) R5P increases the phosphorylation of CDH1 in cells. The phosphorylation levels of overexpressed CDH1 in cells treated with or without R5P‐treated were determined using Phos‐tag SDS‐PAGE. D) R5P and TKTL1 synergistically increase CDH1 ubiquitination by SCF*^β^* ^‐TRCP1^. A commercial ubiquitination system was employed to detect the ubiquitination of purified CDH1 by SCF*^β^* ^‐TRCP1^. Ubiquitination of CDH1 was detected when R5P, recombinant TKTL1, or TKTL1^MUT^ was included, and R5P and recombinant TKTL1 or R5P and TKTL1^MUT^ were present in the reaction mix. E–I) R5P‐bound TKTL1 has stronger interactions with CDH1 and *β*‐TRCP1. Recombinant CDH1, TKTL1, TKTL1MUT, and *β*‐TRCP1 were purified from HeLa cells. The ability of TKTL1 and TKTL1^MUT^ to pull down CDH1 (E) or *β*‐TRCP1 (F) was determined in vitro. The interactions between TKTL1 and CDH1 (G,H) and between TKTL1 and *β*‐TRCP1 (I) were also detected via co‐immunoprecipitation and compared between HeLa cells with or without R5P supplemental. J) R5P induces CDH1‐*β*‐TRCP1 interaction in a TKTL1‐dependent manner. Effects of R5P supplementation on CDH1‐*β*‐TRCP1 interaction were detected by immunoprecipitation in HeLa and *TKTL1* KO HeLa cells. K) TKTL1 D‐box mediates R5P‐facilitated CDH1‐TKTL1 interaction. Interactions between CDH1 and TKTL1 and between CDH1 and TKTL1^△D‐Box^ were detected via co‐immunoprecipitation in HeLa cells with or without R5P treatment. L) TKTL1 SGS mediates R5P‐facilitated *β*‐TRCP1‐TKTL1 interaction. Interactions between *β*‐TRCP1 and TKTL1 and between *β*‐TRCP1 and TKTL1^△SGS^ were detected via co‐immunoprecipitation in HeLa cells with or without R5P treatment. M) Schematic diagram of R5P binding to TKTL1 to facilitate CDH1 phosphorylation by CDK2 and CDH1 recruitment by *β*‐TRCP1.

R5P induces a decrease in CDH1 levels in the G1, S, and early G2/M phases, during which *β*‐TRCP1, the E3 ligase of the SCF*^β^* ^‐TRCP^ complex that targets CDH1 for degradation,^[^ ^30^ ^]^ is expressed (Figure 2K). This suggests that R5P‐mediated CDH1 degradation occurs via SCF*^β^* ^‐TRCP^. TKTL1, which binds R5P (Figure S4A,B, Supporting Information),^[^ ^22^ ^]^ may serve as a sensor for R5P. Disruption of R5P binding to TKTL1 through mutation of His46, His232, Arg292, Ser319, His390, Asp398, and Glu488 to alanine (TKTL1^mut^), which are predicted R5P binding sites of TKTL1 from Uniprot database (Figure S4C, Supporting Information) abrogated the ability of TKTL1 overexpression to decrease CDH1 (**Figure** **4**A), suggesting that R5P binding to TKTL1 is critical for TKTL1 to decrease CDH1 levels. Additionally, R5P and TKTL1 increased CDH1 phosphorylation by CDK2, a prerequisite for CDH1 proteasomal degradation by SCF*^β^* ^‐TRCP^,^[^ ^31^ ^]^ both in vitro (Figure 4B) and in cells (Figure 4C). In a reconstituted ubiquitination assay, R5P and TKTL1 enhanced CDH1 ubiquitination by SCF*^β^* ^‐TRCP^, an effect that was not observed with TKTL1^mut^ (Figure 4D). These findings collectively suggest that R5P binding to TKTL1 promotes CDH1 degradation via SCF*^β^* ^‐TRCP^.

In vitro pull‐down assays demonstrated that neither R5P, TKTL1, nor TKTL1^mut^ alone increased the interaction between CDH1 and *β*‐TRCP (Figure S4D, Supporting Information). However, R5P significantly enhanced TKTL1 binding to both CDH1 (Figure 4E) and *β*‐TRCP1 (Figure 4F). Moreover, TKTL1, but not TKTL1^mut^, facilitated CDH1‐TKTL1 and *β*‐TRCP‐TKTL1 interactions in the presence of R5P (Figure 4E,F; Figure S4D, Supporting Information). R5P supplementation induced both endogenous and overexpression TKTL1 binding to CDH1 and *β*‐TRCP in HeLa cells (Figure 4G–I), but not in *TKTL1^−/−^* HeLa cells (Figure 4J). These results suggest that R5P‐bound TKTL1 bridges CDH1 to *β*‐TRCP for degradation.

TKTL1 contains degrons for both CDH1 and *β*‐TRCP (Figure S4E, Supporting Information)^[^ ^22^ ^]^ which enable TKTL1 to interact with both of them. Disruption of either the CDH1 degron (TKTL1^ΔD‐box^) or the *β*‐TRCP degron (TKTL1^ΔSGS^) of TKTL1 weakened CDH1‐TKTL1 and *β*‐TRCP‐TKTL1 interactions and abrogated the R5P‐facilitated CDH1‐TKTL1 interaction in HeLa cells (Figure 4K,L). These data collectively support that TKTL1 functions as a molecular glue, bridging CDH1 and SCF*^β^* ^‐TRCP^ to mediate CDH1 degradation (Figure 4M). Collectively, these results show R5P binding to TKTL1 facilitates CDH1 degradation by SCF*^β^* ^‐TRCP^.

### 2.5 CDH1 Activates HIF1*α* to Increase Nutrient Supply and Anabolite Production

**Figure 5:**
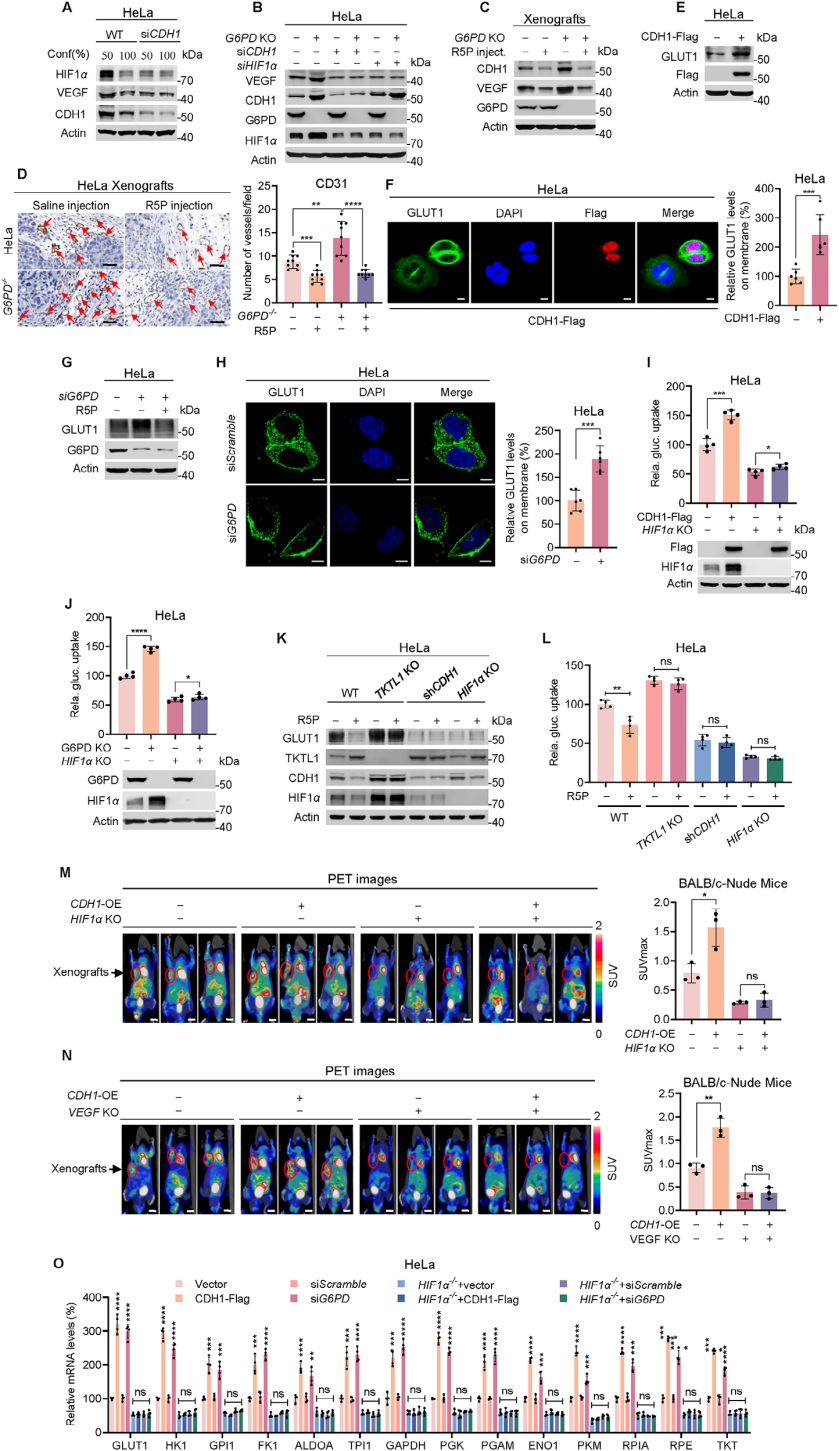
CDH1 promotes angiogenesis and glucose uptake. A) High HIF1*α* and VEGF in proliferating CDH1‐intact cells. Protein levels of HIF1*α* and VEGFA were detected in exponentially growing and stationary stage HeLa and *CDH1*‐silencing HeLa cells. B) G6PD deletion augments VEGF in CDH1‐ and HIF1*α*‐intact cells. Effects of *G6PD* knockout on VEGF protein levels were detected in HeLa and *CDH1*‐, *HIF1α*‐silencing HeLa cells. C,D) R5P decreases angiogenesis. HeLa and G6PD KO HeLa xenografts were grown in BALB/c nude mice intraperitoneally. Mice were fed with or without R5P gavage. The levels of VEGF and CDH1 (C) and vascularization, detected with CD31 staining with quantitation at right (D, *n* = 10), were detected for xenografts. Scale bar, 50 µm. E,F) CDH1 increases GLUT1 expression and membrane localization. GLUT1 protein levels (E) and membrane localization (F, *n* = 6) were detected in HeLa and CDH1‐overexpressing HeLa cells. Scale bar, 10 µm. Quantification is shown on the right. G,H) G6PD silencing increases GLUT1 expression and membrane localization. GLUT1 protein levels (G) and membrane localization (H) were detected in HeLa and *G6PD*‐silenced HeLa cells (*n* = 6). Scale bar, 10 µm. Quantification is shown on the right. I,J) CDH1 and R5P regulate 2‐DG uptake via HIF1*α*. The 2‐DG uptake‐promoting effects of CDH1 overexpression (I, *n* = 4) and *G6PD*‐knockout (J, *n* = 4) were measured in HeLa and *HIF1α* KO HeLa cells. K,L) R5P regulates glucose uptake dependent on TKTL1, CDH1, and HIF1*α*. GLUT1 levels (K) and 2‐DG uptake (L, *n* = 4) were detected in HeLa, *TKTL1* KO HeLa, *CDH1*‐silencing HeLa, and *HIF1α* KO HeLa cells. M,N) CDH1 HIF1*α*‐ and VEGFA‐dependently enhance PET‐CT signal. Effects of CDH1‐overexpression on PET‐CT signal of HeLa xenografts were compared between HeLa and *HIF1α* KO HeLa xenografts (M) and between HeLa and *VEGF* KO HeLa xenografts (N). Quantitation of PET‐CT images is shown at right (*n* = 3). Scale bar, 5 mm. O) CDH1 and G6PD knockdown HIF1*α*‐dependently increase the transcription of GLUT1 and glycolytic/PPP enzyme genes. mRNA levels of GLUT1 and glycolytic/PPP enzyme genes were determined in HeLa and *HIF1α* KO HeLa cells with CDH1 overexpression or *G6PD* silencing (*n* = 4). All data are presented as mean ± S.E.M. Statistical significance was assessed using two‐way ANOVA (D,I,J,L,M,N,O) and two‐tailed unpaired Student's *t*‐test (F,H). *p < 0.05, **p < 0.01, ***p < 0.001, ****p < 0.0001; ns, not significant.

Exponentially growing cells exhibited higher HIF1*α* protein levels and *VEGF* mRNA levels, compared to confluent cells. However, silencing *CDH1* abolished these differences (**Figure** **5**A; Figure S5A,B, Supporting Information), suggesting that HIF1*α* signaling is regulated by CDH1. This hypothesis is further supported by the finding that *G6PD* deletion, which reduces R5P levels, elevates VEFG in a CDH1‐ and HIF1*α*‐dependent manner (Figure 5B). Additionally, R5P inhibited CDH1 and VEGF expression in both HeLa and *G6PD^−/−^* HeLa xenografts (Figure 5C), suggesting that low R5P levels in proliferating cells promote angiogenesis. Indeed, *G6PD* KO HeLa xenografts exhibited higher vascularization (Figure S5C–F, Supporting Information) and increased expression of the vascular differentiation marker CD31^[^ ^29^ ^]^ (Figure 5D), compared to WT HeLa xenografts.

Intraperitoneal injection with R5P, which doubled circulating R5P levels in mice (Figure S5G, Supporting Information), inhibited vascularization in both HeLa and *G6PD^−/−^* HeLa xenografts (Figure 5D). This effect was abolished by *CDH1* silencing (Figure S5H–L, Supporting Information), suggesting R5P inhibited vascularization through decreasing CDH1.

CDH1 overexpression increased the protein levels (Figure 5E; Figure S5M, Supporting Information) and membrane localization (Figure 5F) of GLUT1, both of which are activated by HIF1*α*. *G6PD* knockdown, which decreased R5P levels, also stimulated CDH1 overexpression, promoting GLUT1 expression (Figure 5G) and membrane localization (Figure 5H; Figure S5N, Supporting Information). These results align with the observation that CDH1 overexpression or *G6PD* KO enhanced 2‐deoxyglucose (2DG) uptake in HeLa cells in an HIF1*α*‐dependent manner (Figure 5I,J), which also correlated with changes in *GLUT1* transcriptional levels (Figure S5O, Supporting Information). Furthermore, the loss of *TKTL1*, *CDH1*, or *HIF1α* prevented R5P from decreasing GLUT1 protein levels (Figure 5K) and 2DG uptake (Figure 5L). Positron emission tomography (PET) analysis revealed higher ^18^F‐fluorodeoxyglucose (FDG) signals in CDH1‐overexpressing HeLa xenografts, while deletion of *HIF1α* or *VEGF* resulted in lower FDG signals and abrogated the enhancement of PET signals by CDH1 overexpression (Figure 5M,N). Finally, CDH1 overexpression or *G6PD* knockdown increased the transcription of glycolytic/PPP enzymes in HeLa cells in an HIF1*α*‐dependent manner (Figure 5O). These results collectively confirm that CDH1, or low R5P‐induced CDH1 expression, activates HIF1*α* to increase nutrient supply and anabolic metabolism.

### 2.6 CDH1 Activates AARS2 and Mitochondrial Lactylation to Reserve Anabolites

**Figure 6:**
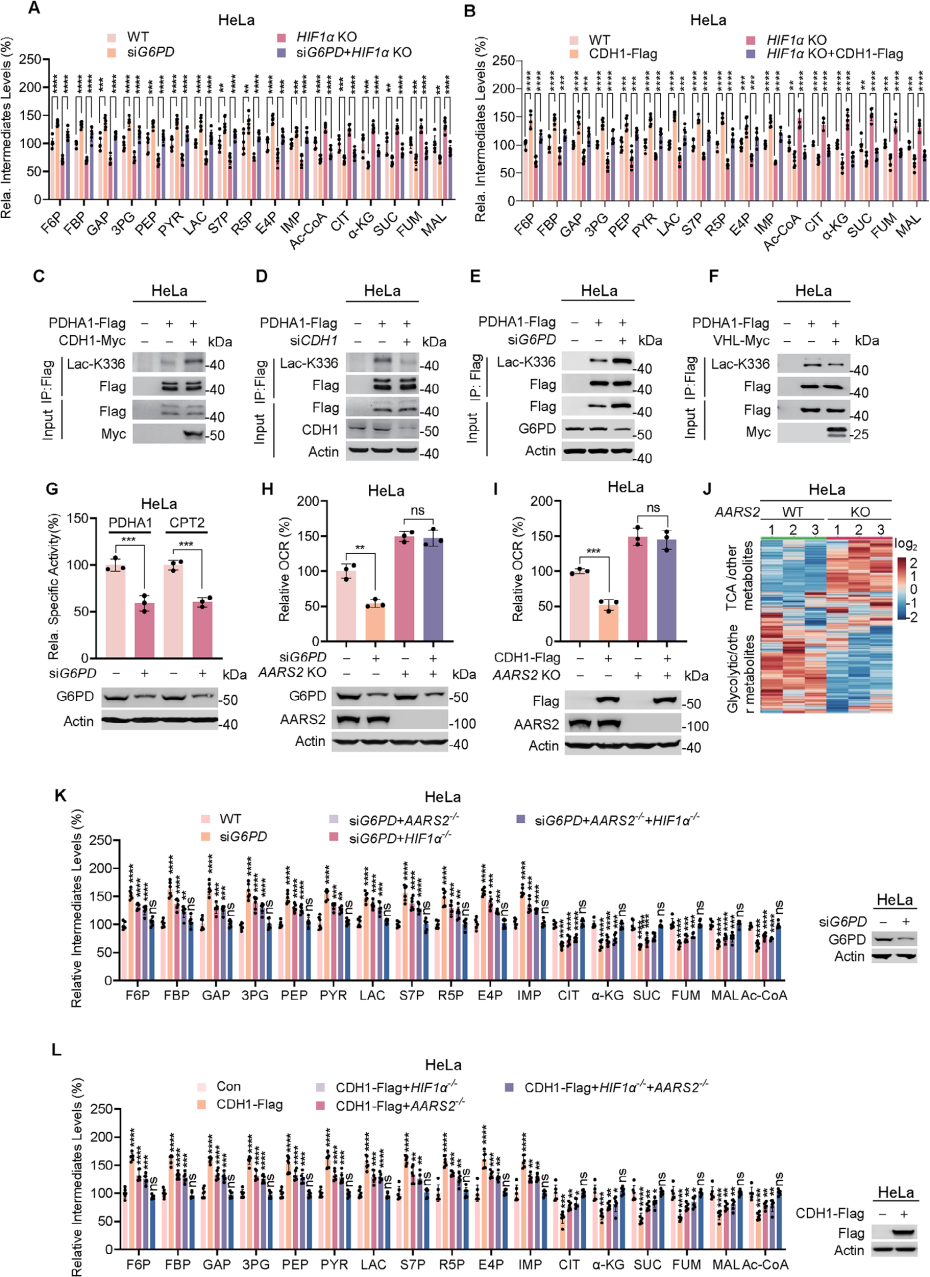
CDH1 induces AARS2 to reserve anabolites. A,B) R5P and CDH1 affect anabolites in *HIF1α* KO HeLa cells. Glycolytic/PPP and TCA metabolites (*n* = 6) were detected in HeLa cells and *HIF1α* KO HeLa cells without or with *G6PD*‐silencing (A) or CDH1 overexpression (B). C–F) CDH1, VHL, and R5P regulate Lac‐K336. Lac‐K336 levels were detected for ectopically expressed PDHA1 in HeLa cells with CDH1 overexpression (C) or *CDH1* silencing (D) and with *G6PD* silencing (E) or VHL overexpression (F). G) *G6PD*‐silencing inactivates PDHA1 and CPT2. Relative (to that of HeLa cells) specific activities of recombinant PDHA1 and CPT2 from HeLa and *G6PD* knockdown HeLa cells were determined (*n* = 3). H,I) G6PD and CDH1 AARS2‐dependently regulate oxygen consumption. Effects of *G6PD* silencing (H, *n* = 3) or CDH1 overexpression (I, *n* = 3) on OCR were determined in HeLa cells and *AARS2* KO HeLa cells. J) AARS2 regulates glycolytic and TCA metabolites. Unbiased metabolomics were performed for HeLa and *AARS2* KO HeLa cells. The changes (red, increased; blue, decreased) in glycolysis/PPP‐associated metabolites and TCA cycle‐associated and other metabolites were compared (*n* = 3) and expressed using a heatmap. K,L) CDH1 and G6PD regulate anabolites accumulation in a HIF1*α*‐ and AARS2‐dependent manner. Effects of *G6PD*‐silencing (K, *n* = 6) or CDH1 overexpression (L, *n* = 6) on levels of glycolytic/PPP and TCA metabolites were detected in HeLa, *HIF1α* KO HeLa, and *HIF1α/AARS2* double KO HeLa cells. OCR, oxygen consumption rate. All data are presented as mean ± S.E.M. Statistical significance was assessed using two‐way ANOVA (A,B,G,H,I,K,L). *p < 0.05, **p < 0.01, ***p < 0.001, ****p < 0.0001; ns, not significant.

CDH1 overexpression or *G6PD* knockdown elevated anabolite levels in *HIF1α*‐deficient HeLa cells (**Figure** **6**A,B), suggesting that CDH1 and low R5P‐induced anabolite accumulation may involve mechanisms independent of HIF1*α* activation. Consistent with this, the CDH1 substrate VHL degraded AARS2, a mitochondrial lactyltransferase that lactylates and inactivates PDHA1 and CPT2, thereby inhibiting the TCA cycle (Figure S6A, Supporting Information).^[^ ^18^ ^]^ CDH1 overexpression increased (Figure 6C; Figure S6B, Supporting Information) and *CDH1* knockdown decreased PDHA1 and CPT2 lactylation (Figure 6D; Figure S6C, Supporting Information). Furthermore, *G6PD* silencing, which increases cellular R5P levels, mimicked the effects of CDH1 overexpression on PDHA1 and CPT2 lactylation (Figure 6E; Figure S6D, Supporting Information). Additionally, VHL overexpression, which is induced by high R5P or low CDH1 levels, decreased PDHA1 and CPT2 lactylation (Figure 6F; Figure S6E, Supporting Information). These results, combined with the finding that increased PDHA1 and CPT2 lactylation induced by *G6PD* silencing resulted in their low specific activities (Figure 6G), but did not affect PDHA1 phosphorylation (Figure S6F, Supporting Information), suggest that CDH1 and low R5P levels promote AARS2‐mediated mitochondrial lactylation to prevent anabolites from being oxidized through the TCA cycle. Supporting this hypothesis, *G6PD* silencing and CDH1 overexpression decreased the oxygen consumption rate (OCR) of HeLa cells in an AARS2‐dependent manner (Figure 6H,I; Figure S6G,H, Supporting Information).

The role of AARS2 in anabolite preservation was further corroborated by increased TCA intermediate levels, decreased glycolysis intermediates/anabolites in *AARS2^−/−^* HeLa Cells (Figure 6J). And pronounced anabolite accumulation with reduced TCA intermediates was observed in cells with HIF1*α*‐ and AARS2‐activating manipulations, such as *G6PD* knockdown (Figure 6K) and CDH1 overexpression (Figure 6L). The absence of *HIF1α* and *AARS2* completely abrogated the anabolite accumulation (Figure 6K,L). Together, these findings suggest that CDH1 or low R5P levels activate AARS2 to preserve anabolites.

### 2.7 Artificial R5P Signal Promotes Cell Cycle Initiation and Cell Apoptosis

**Figure 7:**
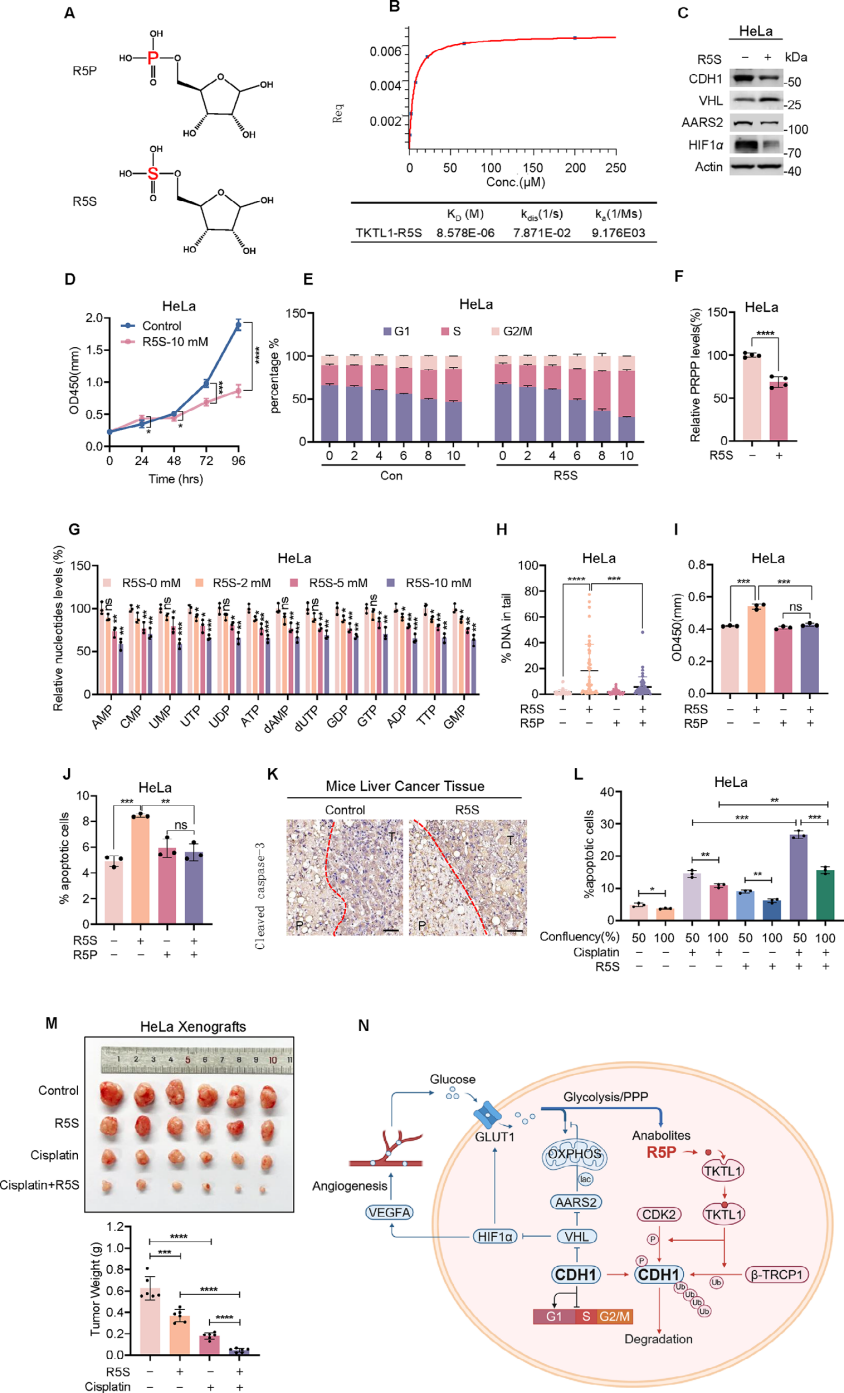
Pseudo R5P signal sensitizes cancer to chemotherapy. A) Chemical structures of R5P and R5S. B) R5S binds to TKTL1. The binding of R5S to recombinant TKTL1 was assayed using a biolayer interferometry (BLI) assay. The binding affinity was determined. C) R5S regulates CDH1, VHL, and HIF1*α* levels. The levels of CDH1, VHL, and HIF1*α* were determined in HeLa and 10 mm R5S‐treated HeLa cells. D) R5S treatment decreased the cell proliferation rate. Growth curves of HeLa and R5S‐treated HeLa cells were measured using a CCK‐8 assay kit (*n *= 6). E) R5S speeds up the G1/S transition. The numbers of cells in the G1, S, and G2/M phases of HeLa cells were detected under the absence and presence of R5S in the media (*n* = 3). F) R5S inhibited PRPP formation. Levels of PRPP were measured in HeLa cells with or without 10 mm R5S treatment (*n* = 4). G) R5S dose‐dependently decreased nucleotide levels. Levels of nucleotides were detected in HeLa cells cultured in the presence of the indicated levels of R5S (*n* = 3). H,I) R5P rescues R5S‐induced DNA damage and AP sites. The levels of DNA damage were measured using the Comet assay (H, *n* = 50) or the AP site assay (I, *n *= 3) in HeLa cells cultured in the presence of R5S or R5P alone, or both. The representative image was shown in Figure S7A, Supporting Information. J) R5P rescues R5S‐induced apoptosis. Apoptotic rates of HeLa cells in HeLa cells cultured in the presence of R5S or R5P alone, or both (*n* = 3). K) R5S induces more pronounced apoptosis in liver cancer tissue. Levels of cleaved caspase‐3 were compared between mouse liver cancer tissue and its adjacent non‐cancer tissue. Scale bar, 50 µm. The quantification and more images were presented in Figures S7B,C, Supporting Information. L) R5S sensitizes cisplatin‐induced apoptosis in proliferating HeLa cells. Effects of cisplatin on apoptotic rates in proliferating HeLa cells and confluent HeLa cells were measured for cells with or without R5S treatment (*n* = 3). M) R5S sensitizes cisplatin to inhibit xenograft growth. The volume (left) and weight (right) were compared among untreated, R5S‐ or cisplatin‐treated, and R5S and cisplatin co‐treated HeLa mouse xenografts (*n* = 6). Food intake and body weight were shown in Figures S7F,G, Supporting Information. N) Schematic representation of how CDH1 accumulates and senses anabolites. CDH1 activates HIF1*α* to activate angiogenesis and glucose uptake to increase R5P production and activates AARS2 to preserve anabolites and R5P. Conversely, R5P feedback inhibited CDH1 by promoting its phosphorylation and degradation. All data are presented as mean ± S.E.M. Statistical significance was assessed using two‐tailed unpaired Student's *t*‐test (F) and two‐way ANOVA (D,G,H,I,J,L,M). *p < 0.05, **p < 0.01, ***p < 0.001, ****p < 0.0001; ns, not significant.

To further confirm that R5P acts as an anabolite‐sufficient signal for CDH1 degradation and cell cycle initiation, we hypothesized that a pseudo‐R5P signal could drive cell cycle initiation without providing sufficient R5P for *de novo* nucleotide synthesis. To test this, we synthesized an R5P analog, ribose‐5‐sulfate (R5S) (**Figure** **7**A). R5S bound to TKTL1 (Figure 7B), and it decreased CDH1, increased VHL, and reduced AARS2 and HIF1*α* levels in HeLa cells (Figure 7C), confirming that R5S mimics R5P signaling.

Supplementing culture media with 10 mm R5S inhibited HeLa cell growth (Figure 7D) but promoted G1/S transition in serum‐deprived, synchronized HeLa cells (Figure 7E). However, R5S inhibited PRPP formation (Figure 7F) and decreased nucleotide levels in a dose‐dependent manner (Figure 7G), implying that R5S induces DNA damage (Figure 7H; Figure S7A, Supporting Information) and the formation of abasic (AP) sites (Figure 7I), all of which are reversible by R5P in HeLa cells. This provides an explanation for why R5S induces apoptosis in HeLa cells in an R5P‐reversible manner (Figure 7J).

The apoptosis‐inducing effects of R5S were further assessed in vivo. In diethylnitrosamine (DEN)‐induced mouse liver cancer, R5S induced significantly more pronounced apoptosis in cancer tissues compared to adjacent non‐cancerous tissues (Figure 7K; Figure S7B,C, Supporting Information), suggesting that the pseudo‐R5P signal generated by R5S is a potent apoptosis inducer in proliferating cells, where *de novo* nucleotide synthesis is active. We hypothesized that DNA‐damaging chemotherapeutic agents could inhibit proliferating cancer cell growth in synergy with R5S treatment. Indeed, cisplatin or 5‐FU co‐treatment with R5S sensitized proliferating HeLa cells to chemotherapy‐induced apoptosis (Figure 7L; Figure S7D, Supporting Information), and this sensitization was also observed in HeLa xenografts (Figure S7E, Supporting Information). Furthermore, R5S showed synergistic effects with cisplatin in inhibiting HeLa xenograft growth (Figure 7M; Figure S7F,G, Supporting Information), highlighting the potential of R5S as a therapeutic strategy for cancer inhibition. Taken together, these data support that R5S provides a pseudo R5P sufficient signal to trigger cell cycle initiation, allow cells to enter irreversible cell cycle without sufficient building blocks for nucleotide synthesis, making cancer cells prone to cisplatin‐induced apoptosis.

## 3 Discussion

Before initiating a new cell cycle, cells must meet specific prerequisites to avoid the detrimental consequences of premature cell cycle initiation, such as apoptosis, since the cell cycle becomes irreversible once initiated.^[^ ^32^ ^]^ To safeguard cell cycle initiation and progression, cells have evolved a complex regulatory network. In the present study, we discovered that, beyond regulating the phosphorylation and protein stability of cell cycle regulators, CDH1, which halts cells in the G1 phase before crossing the G1/S boundary, also plays an important role in regulating anabolites. CDH1 facilitates the degradation of VHL to accumulate anabolites by activating HIF1*α*, which enhances angiogenesis to increase nutrient transport, promotes GLUT1 membrane localization to enhance glucose uptake, and stimulates anabolite production by upregulating glycolytic and PPP enzymes; and activating AARS2, which lactylates and inactivates PDHA1,^[^ ^18^ ^]^ preserving anabolites by inhibiting oxidation via OXPHOS. Interestingly, cells utilize VHL, which regulates cell cycle regulators, to control anabolites, thus synchronizing the anabolite signal with cell cycle regulators during cell cycle initiation.

R5P, the starting anabolite for *de novo* nucleotide synthesis and the first major anabolic process in the cell cycle^[^ ^33^ ^]^ binds to TKTL1 to facilitate the kinase and E3 ligase activities of CDH1, promoting its degradation, a necessary step for initiating a new cell cycle. By regulating both the accumulation and sensing of anabolite sufficiency, CDH1 coordinates anabolites for cell cycle initiation. CDH1 also coordinates the anabolite sufficiency signal to cell cycle initiation factors, which are under the regulation of CDH1. Together, the CDH1‐VHL‐R5P‐CDH1 regulatory circuit ensures that cells enter the new cell cycle only when anabolites are sufficient (Figure 7N). This also explains perturbation in such cell cycle regulators as VHL, which is missing in 80% of renal cancers, or arresting cell cycle with DTB, would cause different R5P changes in our previous and current studies.^[^ ^22^ ^]^

These findings provide insights into the intrinsic drivers of the Warburg effect, which is characterized by enhanced aerobic glycolysis and partially inhibited OXPHOS in cancer cells. CDH1 not only promotes aerobic glycolysis but also inhibits OXPHOS and promotes angiogenesis for nutrient transport, a phenomenon commonly observed in cancer. Thus, CDH1 drives reprogramming beyond the Warburg effect. The multiple roles of CDH1 may explain some of the puzzling observations about this protein. Despite being considered a tumor suppressor because it halts cells in the G1 phase, CDH1 loss‐of‐function mutations have not been observed in cancers.^[^ ^13^ ^]^ In contrast, CDH1 is frequently overexpressed in malignant tumors.^[^ ^34^ ^]^ This paradox likely arises because R5P levels are low in proliferating cells, which prevents the TKTL1‐mediated degradation of CDH1. The roles of CDH1 in metabolic reprogramming may also explain why the Warburg effect and other metabolic changes are observed not only in cancer cells but also in proliferating non‐cancerous cells.

Our discovery that R5P levels represent an indicator of anabolite sufficiency opens up alternative strategies for cancer treatment through metabolic intervention. We demonstrated that a pseudo‐R5P signal, generated by the R5P analog R5S, sensitizes cancer cells to chemotherapy‐induced apoptosis by prematurely initiating the cell cycle with insufficient anabolites. Further studies are warranted to confirm this intervention, as our results have not yet been verified in patients.

## 4 Experimental Section

### Ethics Statements

Human materials used in this study were approved by the Ethics Committee of the Obstetrics and Gynecology Hospital of Fudan University (Approval number: 2022–33). All animal experimental protocols were approved by the Ethics Committee of Experimental Animals of Fudan University (Approval number: 2022JS‐026).

### Clinical Samples

Six human cervical cancer tissues (Patient information in Table S1, Supporting Information) were obtained from Obstetrics and Gynecology Hospital of Fudan University with the signed consent from the patients.

### Animals

All animal procedures were performed in accordance with the guidelines of the animal care committee at Fudan University. BALB/C nude mice and C57BL/6J mice were purchased from Shanghai SLAC Laboratory Animal Co., Ltd. All mice were housed in a specific pathogen‐free facility at 20–22 °C on a 12 h light/dark cycle with ad libitum access to food and water. For the xenograft study, Athymic (BALB/c, nu/nu, 6‐week‐old) male, nude mice were obtained, and the mice were housed in autoclaved plastic filter‐top cages, maintained in a laboratory animal facility with ad libitum access to water, and acclimatized for at least 1 week before the beginning of the study. The mice were randomly put into separate cages for experiments and received a standard chow diet. Xenografts were established from the wild‐type and gene‐modified HeLa cells by the subcutaneous implantation of 2 × 10^6^ cells in PBS into the flank of each animal. Tumor volume was measured by using length (a) and width (b) with Vernier calipers and calculated using the equation: *V* = ab^2^/2. When tumors reach significant sizes (average of 100–200 mm^3^), R5S (3 mg kg^−1^), R5P (3 mg kg^−1^), and cisplatin (1 mg kg^−1^) were administered to mice every other day through intraperitoneal injection. Tumor volume was measured every other day for 2 weeks. All tumors were harvested at the indicated times and weighed.

### Cell Lines and Cell Culture

Human cervix cancer HeLa cells, human cervical squamous cell carcinoma SiHa cells, and human cervical immortalized squamous Ect1/E6E7 cells were purchased from American Type Culture Collection (ATCC). Cell lines were tested and authenticated by morphology and growth rate, and were confirmed to be mycoplasma‐free. Cells were grown in Dulbecco's modified Eagle's medium (DMEM, Gibco) supplemented with 10% fetal bovine serum at 37 °C with 5% CO_2_ in a humidified incubator.

### Plasmid

CDH1, CDC20, cyclinA2, TKTL1, VHL, PDHA1, and CPT2 were cloned into pcDNA3.1(b+)‐Flag, ‐HA or ‐Myc between XhoI and EcoRI. SKP2 and *β*‐TRCP1 were cloned into pRK7 between HindIII and EcoRI. TKTL1 R5P‐binding sites mutant was constructed by simultaneously switching His46, His232, Arg292, Ser319, His390, Asp398, and Glu488 all into alanine. The TKTL1^△D‐box^ plasmid was constructed by simultaneously switching both Arg18 and Leu21 to alanine, the TKTL1^△SGS^ plasmid was constructed by simultaneously switching both Ser564 and Gly565 to alanine. The CDH1^MUT^ plasmid was constructed by simultaneously switching Ser40, Thr121, Ser151, Ser163 to alanine.

### De Novo Purine Synthesis Inhibition

*De novo* purine synthesis inhibitor mizoribine and *de novo* pyrimidine synthesis inhibitor teriflunomide were dissolved in dimethyl sulfoxide (DMSO) as 100 mm stock. These two inhibitors were simultaneously added into the culture medium to reach a final concentration of 50 µm for 6 h before nucleotide detection.

R5P or R5S was added to culture media at a final concentration of 10 mm for 6 h before harvest.

### Cell Cycle Synchronization

Thymidine synchronization: Adherent 20–30% confluent HeLa cells were cultured in DMEM containing 2 mm thymidine for 18 h before rinsing twice with PBS, and replaced with thymidine‐free DMEM. After 9 h, cells were reincubated with DMEM containing 2 mm thymidine for 18 h. Release of blocked cells was achieved by removing thymidine from the media. The cells were sampled at the indicated times and subjected to flow cytometric analysis before other measurements. Serum deprivation synchronization: removing serum from the medium for 48 h to arrest the cells in G0 phase, the releasing was achieved by the addition of serum back to the medium to stimulate cell cycle entry into the early G1 phase.

### Cell Transfection

For plasmid transfections, the encoding plasmids were performed using polyethylenimine (PEI, Polyscience) or Lipofectamine 8000 (Beyotime). For transfection with PEI, the plasmids and PEI were added to serum‐free DMEM and mixed by vigorous shaking; the mix was then incubated for 15 min before adding to the cell culture medium. For transfection with Lipofectamine 8000, the plasmids and Lipofectamine 8000 were added to serum‐free DMEM, mixed gently by pipetting and then immediately added to the cell culture medium. The medium was replaced with fresh medium at 12–16 h after transfection. Cells were harvested after 36 h.

### Small RNA Interference (RNAi)

The siRNA transfections were performed with Lipofectamine 8000 (Beyotime) according to the manufacturer's instructions. Cells were cultured in 6‐well plates (ThermoFisher Scientific), and siRNAs were transfected at a concentration of 20 nm synthetic siRNA oligonucleotides (Genepharma, Shanghai). For each target gene, at least two effective target sequences were employed to exclude off‐target effects. Knockdown efficiency was verified by quantitative real‐time polymerase chain reaction or western blotting. The siRNA target sequences used in this study are listed in Table S3, Supporting Information.

### Stable Cell Lines

For the establishment of stable overexpression or knockdown HeLa cell lines, a retrovirus system was adapted. Cells were cultured in a 10 cm dish and 2 µg VSVG, 2 µg GAG, and 3 µg pBABE‐CDH1 or pmko‐shCDH1 were co‐transfected using Lipofectamine 8000 (Beyotime). The culture supernatant was collected after 24 and 48 h of transfection, to be used for infection. Then, 40 µL 100× polybrene was added to the medium during infection. After 24 h of infection, 2 µg mL^−1^ puromycin (Amresco) was added to the cells. The knockdown and overexpression efficiency were determined by western blotting 5 days after puromycin selection. The shRNA targeting sequences used are listed in Table S3, Supporting Information.

### Gene Knockout

Knockout cell lines adapted the CRISPR/Cas9 system. The plasmid was constructed by cloning the annealed sgRNA into the px459 vector. The sgRNAs were designed using the CRISPR Design website (http: //crispr.mit.edu). Guide RNAs were transfected into HeLa cells, followed by selection with 2 µg mL^−1^ puromycin (Amresco). After 3 days, the surviving cells were harvested and seeded into a 96‐well plate at a density of 1 cell well^−1^. The knockout efficacies were verified by western blotting. The sgRNA targeting sequences used were listed in Table S3, Supporting Information.

### Glucose Uptake Assay

Glucose uptake was determined using the Glucose Uptake‐Glo Assay (J1342, Promega, USA) according to the manufacturer's instructions. Briefly, HeLa cells were plated at 2 × 10^4^ cells well^−1^ in a 96‐well plate and incubated overnight. The medium was removed and the cells were washed twice with PBS; the cells were then treated with 1 mm 2DG in PBS for 30 min. Then, 25 µL of Stop Buffer followed by 25 µL of Neutralization Buffer was added and shaken briefly. Finally, 100 µL of 2DG6P detection reagent was added, and luminescence was recorded using 0.5 s integration on a luminometer. Measurements were performed for at least four replicates.

### Flow Cytometry

For apoptosis detection, adherent cells were stained using the Annexin V‐Alexa Fluor 488/PI Apoptosis Detection Kit (40 305, Yeason, China) according to the manufacturer's instructions. Briefly, cells were washed twice with cold PBS and then digested with trypsin and centrifuged for 5 min at 1000 rpm. The supernatant was removed, and the cell pellet was suspended in 100 µL 1× Binding Buffer at a concentration of 1 × 10^6^ cells mL^−1^. Then, 5 µL of FITC Annexin V and 5 µL of PI were added and incubated for 15 min, followed by adding 400 µL of 1× Binding Buffer to the sample tube. The cells were analyzed using a fluorescence‐activated cell sorter (FACS Calibur, BD). For cell cycle analysis, cells were digested using trypsin and suspended overnight in cold 70% ethanol. The fixed cells were then incubated in PBS containing RNase A (100 mg mL^−1^) and PI (50 mg mL^−1^) for 30 min at 37 °C and analyzed using a fluorescence‐activated cell sorter (FACS Calibur).

### Oxygen Consumption Rate (OCR)

For OROBOROS Oxygraph‐2K module assays, ≈1 × 10^6^ cells were digested and resuspended in PBS. The basal respiratory rate was measured, and respiration was then inhibited using Oligomycin A (Selleck, China). The oxygen consumption values were normalized to the cell number or protein content.

### Comet Assay

The comet assay was performed using the CometAssay kit (Bio‐techne, MN, USA, Cat # 4250‐050‐K) following the manufacturer's instructions. Briefly, gently detach cells from the flask surface. Transfer cells and medium to the centrifuge tube, perform a cell count, and then pellet cells. Wash once in ice‐cold 1× PBS (Ca^2+^ and Mg^2+^ free). Suspend cells at 1 × 10^5^ cells mL^−1^ in ice‐cold 1 × PBS (Ca^2+^ and Mg^2+^ free). Combine cells at 1 × 10^5^ mL^−1^ with molten LMAgarose (at 37 °C) at a ratio of 1:10 (v/v) and immediately pipette 50 µL onto Com etSlide. The slide was incubated at 4 °C in the dark for 10 min and then transferred to Lysis Solution (Cat # 4250‐050‐01) for 30–60 min at 4 °C. Drain excess buffer from slides and immerse in freshly prepared Alkaline Unwinding Solution, pH > 13 (200 mm NaOH, 1 mm EDTA) at room temperature for 30 min, in the dark. Added ≈850 mL 4 °C Alkaline Electrophoresis Solution (200 mm NaOH, 1 mm EDTA, cool to 4 °C), placed slides in electrophoresis slide tray (slide label adjacent to black cathode), and covered with Slide Tray Overlay. Power supply was set to 21volts and applied voltage for 30 min. Gently drained excess electrophoresis solution, gently immersed twice in ddH_2_O for 5 min each, then in 70% ethanol for 5 min. Samples were dried at 37 °C for 10–15 min. For observation, samples were stained with 100 µL of diluted SYBR Green onto each circle of dried agarose and stained 30 min (room temperature) in the dark, and then viewed slides by epifluorescence microscopy.

### AP Sites Assay

The levels of AP sites were evaluated using the OxiSelect Oxidative DNA Damage Quantitation Kit (Cell Biolabs, Inc., San Diego, CA, USA, Cat#STA‐324) according to the manufacturer's protocol. Genomic DNA (gDNA) was isolated from the cells and dissolved in TE buffer. To quantify AP sites in the genomic DNA of samples, gDNA and aldehyde reactive probe (ARP) were mixed to react specifically with an aldehyde group on the open‐ring form of AP sites, and the ARP was tagged with biotin/streptavidin‐enzyme, which allowed detection of the AP sites by OD 450 nm using the ELISA reader.

### CCK8‐Cell Proliferation Assay

Cell Counting Kit‐8 (CCK‐8) was used to measure cell proliferation. In all, 96‐well plates were seeded with 1 × 10^3^ cells per well. The cells were sampled at 24, 48, 72, and 96 h; the supernatant was removed and 100 µL DMEM containing 10 µL of CCK8 was added into the wells and incubated for 1 h at 37 °C. Absorbance at 450 nm was measured using a plate reader (SpectraMax i3x, Molecular Devices).

### 18^F^‐FDG PET/CT

The animals were fasted for 6 h, followed by 100 µCi 18^F^‐FDG tail vein injection. Mice were maintained under 1 L min^−1^ of 2% isoflurane for anaesthesia during the accumulation and scanning periods. The core body temperature of the animals was maintained using temperature‐controlled cages during the periods of unconsciousness to minimize brown‐fat metabolism from hypothermia. After 1 h accumulation of 18^F^‐FDG, the mice were placed on a warm pad, and a 20‐min emission scan was acquired. All images were acquired using a Siemens Inveon PET/CT scanner at the Department of Nuclear Medicine, Cancer Hospital of Fudan University, China. The PET/CT image analysis was performed as follows: 3D regions of interest were drawn using the trace command around the tumours on scan slices over the tumours and the total activity of all tumors slices was summed. The total activity was divided by the time‐corrected dose‐delivered [time‐corrected dose = dose injected × exp (−0.006317 ×* t*)], in which *t* is the time between the injection and scan time, and is shown as the fold change of the baseline scan of each respective tumor. Representative images were analyzed using Siemens Inveon Research Workplace software (v3.1.2). The percentage injected dose per gram (%ID/g) was calculated as follows: %ID/g = (ROI activity/injected dose) × 100%. The standardized uptake value (SUV) was calculated as follows: SUV = ROI activity × mouse weight/injected dose.

### Mouse Liver Cancer Induction with Diethyl Nitrosamine (DEN)

The body weight of the mice was determined at the time of dosing for the chemical introduced. DEN (25 mg kg^−1^) was administrated intraperitoneally once in 14‐day‐old C57/BL6 male mice. Tumors developed in the liver after ≈30 weeks of administration.

### Mouse R5S Treatment

R5S (3 mg kg^−1^) was administered to mice through intraperitoneal injection once every three days for 6 weeks before liver tumors were harvested by sacrificing the mice.

### SDS‐PAGE and Western Blot

Sodium dodecyl sulphate‐polyacrylamide gel electrophoresis (SDS‐PAGE) and Western blotting were performed following the standard protocols. Briefly, exponentially growing cells on 100 mm plates were washed once with cold PBS and lysed directly in 1 mL SDS loading buffer containing 50 mm Tris‐HCl pH 6.8, 10% glycerol (v/v), 2% SDS (w/v), 4% *β*‐mercaptoethanol (v/v), and 0.0012% bromophenol blue (w/v). For western blot analysis, each sample was subjected to SDS‐PAGE and transferred to nitrocellulose membranes (GE Healthcare Life Science). The membranes were blocked in 5% (w/v) skim milk in Tris‐buffered saline with 0.1% (v/v) Tween‐20 (TBST) for 1 h at RT and were then probed overnight with primary antibodies (see Table S4, Supporting Information) in antibody dilution buffer (QuickBlock, Beyotime) at 4 °C. After incubation with horseradish peroxidase (HRP)‐conjugated secondary antibodies in TBST (containing 5% skim milk), membranes were developed using ECL‐Plus (Thermo Fisher Scientific), visualized using the Typhoon system (GE Healthcare Life Science) and analyzed by Image J.

### Immunoprecipitation

Cells were lysed with 0.5% NP‐40 buffer (50 mm Tris‐HCl pH7.5, 150 mm NaCl, 0.5% NP‐40) supplemented with protease inhibitors which included 1 µg mL aprotinin, 1 µg mL leupeptin, 1 µg mL pepstatin, 1 mm Na_3_VO_4_, 1 mm PMSF, and 10 mm NaF. Whole cell lysates were harvested by centrifugation at 12 000 rpm at 4 °C for 10 min. After centrifugation, Flag beads (Sigma) were added to the supernatant. Slowly rotated the mixture at 4 °C overnight. The next day, the binding complexes were washed at 2000 rpm, 4 °C for three times with ice‐cold 0.5% NP‐40 buffer. Samples were added loading buffer and boiled 10 min at 99 °C for SDS‐PAGE.

### Quantitative RT‐PCR

The ChamQ SYBR qPCR Master Mix kit (vazyme) was used for quantitative RT‐PCR, *β*‐actin was used as the endogenous control for samples. The primer sequences used are listed in Table S5 (Supporting Information).

### Ubiquitination Detection

HeLa cells were co‐transfected with the indicated plasmids for 36 h and then treated with 10 µm MG132 for 6 h before harvesting. Cells were next lysed in ubiquitination lysis buffer (50 mm Tris‐HCl pH 7.5, 1%SDS, 0.5 mm EDTA) containing 1 mm DTT. The lysates were mixed by pipetting up and down and were boiled at 99 °C for 5 min. When the cell pellets became clear, the lysates were diluted with nine times (900 µL) of 0.5% NP‐40 Buffer. Centrifugated at 12 000 rpm at 4 °C for 10 min. Flag Beads (Sigma) were used for immunoprecipitation. Samples were added loading buffer and boiled 10 min for SDS‐PAGE.

### In Vitro CDH1 Ubiquitination

CDH1 was ubiquitinated in a 15 µL reaction by incubation with E1 (40 nm, Biotech, Cat# E‐305‐025), E2 (5 µm, Prospec, Cat# ENZ‐355), SCF (50 nm, Ubiquiget, Cat# 63‐1001‐025), *β*‐TRCP1 (50 nm, Proteintech, Cat#Ag28739), and Ub (25 µm, bio‐techne, Cat# U‐100H) in Ub buffer (40 mm Tris‐HCl, pH 7.5, 5 mm MgCl_2_) containing 1 mm DTT and 0.5 mm ATP at room temperature for 1 h. For analysis of the ubiquitination of CDH1 by SDS‐PAGE, the assay mixture was mixed with 5× SDS loading buffer containing 250 mm DTT prior to gel electrophoresis. Western blotting using anti‐HA tag antibody could be used to analyze the ubiquitination of CDH1.

### In Vitro CDK2 Kinase Assay

CDH1 was phosphorylated by CDK2‐cyclin E1 in vitro. CDH1‐Flag was purified from HEK293T cells by immunoprecipitation and washed twice with CDK2 kinase assay buffer before the reaction. The CDK2‐Flag and cyclin E1‐myc were co‐transfected into HEK293T cells and the CDK2‐cyclin E1 complex was purified by immunoprecipitation using flag beads. The CDK2‐cyclin E1 complex was eluted from the beads with CDK2 kinase assay buffer containing 50 µg µL^−1^ Flag peptide and stored on ice until use. CDH1‐Flag was incubated with or without the CDK2‐cyclin E1 complex in 200 µL CDK2 kinase assay buffer at 37 °C for 1 h. The CDK2 kinase assay buffer contained 10 µm ATP, 50 mm HEPES (pH 7.5), 1 mm dithiothreitol, 10 mm MgCl_2_, 0.1 mm Na_3_VO_4_, and 1 mm NaF. The reaction was terminated by adding 5× SDS loading buffer followed by boiling the samples for 10 min. Finally, reaction mixture proteins were resolved by 10% SDS‐PAGE containing 5 mm phos‐tag acrylamide to detect the phosphorylation of CDH1.

### Protein Pull Down

Flag‐tagged and Myc‐tagged plasmids were co‐transfected for 36 h. Cells were lysed with 0.5% NP‐40 buffer (50 mm Tris‐HCl pH7.5, 150 mm NaCl, 0.5%NP‐40) supplemented with protease inhibitors which included 1 µg mL^−1^ aprotinin, 1 µg mL^−1^ leupeptin, 1 µg mL^−1^ pepstatin, 1 mm Na_3_VO_4_, 1 mm PMSF, and 10 mm NaF. Whole cell lysates were harvested by centrifugation at 12 000 rpm at 4 °C for 10 min. After centrifugation, Flag beads (Sigma) were added to the supernatant which contained Flag‐tagged protein for immunoprecipitation at 4 °C for 4 h. The supernatant which contained Myc‐tagged protein was first incubated with anti‐Myc antibody at 4 °C for 30 min and then with protein A beads (Millipore) for 3 h. Peptides (GL biochem) were used to compete for the replacement of Myc‐tagged proteins from protein A beads at 4 °C for 1 h. Incubated Flag‐tagged protein captured by Flag beads and Myc‐tagged protein existing in the supernatant at 4 °C overnight. The next day, the binding complexes were washed at 2000 rpm, 4 °C three times with ice‐cold 0.1% NP‐40 buffer. Samples were added loading buffer and boiled 10 min for SDS‐PAGE.

### PDHA1 Activity Assay

Cells co‐expressed PDHA1‐Flag and PDHB‐Myc were lysed on ice using 0.1% NP‐40 buffer supplemented with protease inhibitors. PDHA1 protein was immunoprecipitated by Flag beads (Sigma Aldrich). PDHB co‐purified with PDHA1 was quantified via western blot using anti‐Myc tag antibody and used to normalize the activity of PDHA1. The PDHA1 activity was performed in the model reaction with 2,6‐dichlorophenolindophenol (2,6‐DCPIP) by monitoring the reduction of the OD values of 2,6‐DCPIP at 600 nm. The reaction buffer containing 50 mm KH_2_PO_4_ (pH 7.0), 1 mm MgCl2, 2 mm sodium pyruvate, 0.2 mm thiamin diphosphate, and 0.1 mm 2,6‐dichlorophenolindophenol (2,6‐DCPIP) was maintained at 30 °C. Adding PDHA1 purified by flag beads into the reaction buffer initiated the reaction. One unit of PDHA1 activity was defined as the amount of 2,6‐DCPIP reduced (µmol min^−1^ mg^−1^).

### CPT2 Activity Assay

CPT2 activity was assayed by measuring the decrease in CoA levels. HEK293T cells expressing CPT2‐Flag were collected following immunoprecipitating using anti‐Flag beads to acquire CPT2 protein. The reactions were conducted in a 300 µL reaction mix containing 50 mm Tris‐HCl (pH 7.5), 120 mm KCl, 1 mm EDTA, 2 mm CoA, and 2 mm Palmitoyl‐L‐Carnitine. To initiate the reaction, 20 µg proteins were transferred into the reaction mix cultured in a thermostatic oscillation incubator at 1000 rpm and 37 °C, and the mix was collected at 0, 5, 10, 20, and 30 min. The reaction was terminated by adding 50 µL of 10 mm DTNB. The supernatant was monitored using a microplate reader at 410 nm.

### G6PDH Activity Assay

G6PDH activity was assayed by kits according to the manufacture's protocol. Approximately 1 × 10^6^ cells were lysed in 200 µL extraction buffer, and the supernatant was collected after centrifugation at 12 000 × g, 4 °C for 5–10 min. Then 50 µL supernatant was incubated with 50 µL G6PDH detection solution at 37 °C protected from light for 10 min. Absorbance at 450 nm was measured.

### Transketolase Activity Assay

Transketolase activity was assayed by kits according to the manufacture's protocol. Approximately 4 × 10^5 ^cells were homogenized with 100 µL TKT assay buffer. The lysates were kept on ice for 10 min followed by centrifugation at 10 000 × g for 15 min at 4 °C. The supernatant was collected to estimate protein concentration. Samples (2–4 µL) were mixed with 50 µL reaction mix. Fluorescence was immediately recorded at 30 s intervals for 30–45 min at 37 °C.

### NADPH Assay

NADPH levels were determined with kits according to the manufacture's protocol. Approximately 1 × 10^6^ cells were lysed in 200 µL extraction buffer, and the supernatant was collected after centrifugation at 12 000 × g, 4 °C for 5–10 min. Then the supernatant was incubated at 60 °C for 30 min to decompose NADP^+^. After that, 50 µL samples were incubated with 100 µL G6PDH working solution at 37 °C protected from light for 10 min. And 10 µL color reagent was added to each well, and the mixture was incubated at 37 °C protected from light for 10–20 min. Absorbance at 450 nm was measured.

### Confocal Microscopy

Cells were plated in glass‐bottom culture dishes at a density of 1 × 10^5^ per well. Cells were fixed with 4% paraformaldehyde for 15 min and then permeabilization with 0.3% Triton X‐100 for 15 min at room temperature. Then the cells were incubated with 5% bovine serum albumin for 1 h at room temperature and followed by incubation with primary antibody overnight at 4 °C and secondary antibody for 1 h at room temperature in the dark. DAPI was incubated away from light at room temperature for 3–5 min. Images were obtained in multi‐tracking mode on a confocal laser scanning microscope (LSM880 Airyscan, Zeiss) with a 40× lens and processed by ZEN2.6 software. The immunofluorescence for GLUT1 was performed under non‐permeable conditions. Cells were fixed with 4% paraformaldehyde for 15 min and then directly incubated with 10% goat serum 1 h at room temperature.

### Immunohistochemical Staining

Paraffin sections were successively placed in xylene I (15 min), xylene II (15 min), xylene III (15 min), alcohol I (5 min), alcohol II (5 min), 85% alcohol (5 min), 75% alcohol (5 min), and distilled water. The sections were then placed in Citrate Antigen Retrieval solution (pH 6.0) and brought to a boil over medium heat for 8 min. After turning off the heat for 8 min, heating was performed over medium‐low heat for 7 min, during which the sections were not allowed to dry out. The sections were then placed in 3% hydrogen peroxide, away from light, for 25 min at room temperature and blocked with 3% BSA for 30 min at room temperature, incubated overnight with primary antibodies in a wet box at 4 °C, and incubated with IgG‐HRP for 50 min at room temperature. Finally, the sections were incubated with freshly prepared DAB reagent, and the reaction was stopped using running water. Counterstaining was performed with haematoxylin for 3 min and then flushed with water. Sections were then successively immersed in 75% alcohol (5 min), 85% alcohol (5 min), alcohol I (5 min), alcohol II (5 min), butanol (5 min), and xylene I (5 min), and were then sealed with neutral balsam. The sections were observed under a microscope, and images were captured.

### Expression and Purification of Recombinant TKTL1 Protein

Expression and purification of recombinant human TKTL1 and TKTL1^mut^ were achieved by cloning the CDS sequence and its mutants of TKTL1 into pET‐22b(+). When the OD600 of LB culture medium reached 0.5–0.6, 0.5 mm IPTG was added into the culture medium to induce protein expression in *E. coli*. BL21(DE3) cells at 16 °C for 14 h. Cells were collected by centrifugation at 4000 rpm for 15 min at 4 °C followed by sonication and filtration through a 0.22 µm membrane filter. Recombinant His‐tagged proteins were then purified by a standard Ni‐NTA purification procedure using HisTrap HP (GE Healthcare) and an purification AKTA Pure system (GE Healthcare). The eluted proteins were then centrifuged by Amicon Ultra‐15 (Millipore, USA) to remove imidazole and concentrate proteins. Protein samples were then quantified using a spectrophotometer by measuring absorbance at 260/280 nm and the BCA assay (Beyotime Biotechnology). To assess purity, protein samples were run on SDS‐PAGE and stained with Coomassie blue. Proteins were stored in 50 mm Tris‐HCl containing 20% glycerol at −80 °C for BLI experiments.

### Protein Thermal Stability Assay

Cultured cells were treated with 5 mm R5P for 6 h, and then lysed in ice‐cold PBS containing a protease inhibitor cocktail by repeated liquid nitrogen freeze–thaw. For in vitro treatment, cells were first lysed as described above, and the lysates were incubated with 5 mm R5P for 6 h. Both sets of samples were then subjected to a thermal gradient in a thermocycler (46–76 °C, 6 °C increments, 3 min per step), immediately cooled on ice, and centrifuged at 20 000 × g for 20 min at 4 °C. Equal volumes of the supernatants were collected and mixed with SDS loading buffer for western blot.

### BLI (Biolayer interferometry)

Binding affinities of R5P, R5S to TKTL1 and TKTL1^mut^ were determined by BLI using an Octet RED96 system (Sartorius). Anti‐his biosensors were used for initial his‐tagged TKTL1 protein loading. Anti‐His coated biosensors were pre‐hydrated in PBST buffer for 10 min and then transferred to microplate wells containing his‐tagged TKTL1 or TKTL1^mut^ protein at a concentration of 20 µg mL^−1^ for immobilization for 10 min. Baseline BLI measurements were carried out for 60 s in PBST buffer. Association rate were measured by transferring the biosensors to wells supplemented with R5P or R5S that were threefold serially diluted over a range of 0.82–200 µm for 60 s. Dissociation was then measured for 180 s by transferring the biosensors back to the wells used for baseline recording. Non‐specific binding of R5P or R5S to the biosensor surface was subtracted by referencing against biosensors with no TKTL1 or TKTL1^mut^ protein immobilized. Data acquisition was performed at 30 °C. Data were analyzed using the Octet BLI Analysis software 12.2. The data were fit to a 1:1 binding model to calculate an association and dissociation rate, and KD was calculated using the ratio kd/ka.

### LC‐MS/MS Metabolites Detection

Approximately 1 × 10^7^ cells were treated with a cold aqueous methanol solution (80% v/v) to quickly stop cellular metabolism. Cells were scraped off into tubes and placed overnight at −80 °C. Samples were centrifuged at 15 000 × *g* for 15 min at 4 °C, and supernatants were collected. The supernatants were then lyophilized and redissolved in 500 µL of methanol/water (10:90 v/v). The separated metabolites were acquired using high‐performance liquid chromatography (HPLC) employing an LC‐20AB pump (Shimadzu, Kyoto, Japan) and the Luna NH2 column (P/N 00B‐4378‐B0; 5 µm, 50 × 2 mm; Phenomenex, Torrance, CA). The sample injection volume was 2 µL. The mobile phase comprised eluent A (0.77 g NH_4_OAc, 1.25 mL NH_4_OH, 25 mL ACN, and 300 µL acetic acid [HAc] dissolved in 500 mL water) and eluent B (ACN). The elution program was as follows: 0.1 min, 85% B; 3 min, 30% B; 12 min, 2% B; 15 min, 2% B; and 16–28 min, 85% B. The flow rate of the pump was 0.3 mL min^−1^, and the mass spectrometer used was the 4000 QTRAP system (AB Sciex, Framingham, MA) operated in the multiple reaction monitoring mode. The MS parameters were electrospray voltage, 5 kV; gas 1, 30; gas 2, 30; curtain gas, 25; and temperature, 500. Metabolites were monitored for the following ions: F6P 259–97; FBP 339–97; GAP 169–97; 3PG 185‐79; PEP 167–79; PYR 87‐43; LAC 89‐43; CIT 191‐110; AKG 145‐101; SUC 117–73; FUM 115–71; MAL 133‐115; Acetyl‐CoA 810–60; IMP 347–79; ATP 509–79; S7P 289–97; R5P 229–97; and E4P 199‐79. Each measurement was obtained at least in triplicate. Raw data were converted to the mzXML format using ProteoWizard and processed using an in‐house programme developed using R and based on XCMS, for peak detection, extraction, alignment, and integration. An in‐house MS2 database (BiotreeDB) was applied for metabolite annotation. The cut‐off for annotation was set at 0.3.

### Blood Vessels Quantification

IHC staining of CD31 was performed to quantify blood vessels. Three sections from each tissue specimen were prepared and assayed. For each section, three microscopic fields were randomly sampled at 20× magnification, and the number of blood vessels was evaluated.

### DESI‐MS and DESI‐MS Imaging

Tumor tissue and adjacent non‐tumor tissues were embedded in cellulose monocarboxylic acid (CMC) and sectioned. Adjacent sections were reserved for hematoxylin and eosin (H&E) staining to guide region‐of‐interest (ROI) annotation. The imaging experiment was performed on a Quadrupole cIM Time‐of‐Flight (Cyclic IMS) MS Spectrometer equipped with a Desorption ElectroSpray Ionization (DESI) XS source (Waters, Milford, MA, USA). The parameters were optimized for the best signal intensity of R5P: capillary voltage 1 kV, cone voltage 40 V, source temperature 120 °C, and nitrogen gas pressure 0.09 MPa. MeOH is used as a solvent, and the flow rate was 2 µL min^−1^. Pixel size was 100 µm × 100 µm and scan rate was 200 µm s^−1^. Full scan mass spectra were acquired in negative mode with the mass range of m/z 50–1200. Imaging data were acquired in the MassLynx v4.2 platform with an endogenous ion m/z 255.2324 as a lock mass to do mass correction. HDI v1.6 was used to transfer mass spectral data to 2D spatially resolved ion images. Ion images of target metabolites in PPP and fatty acids, which were set as controls, were generated. The absolute intensity values were extracted from annotated ROIs corresponding to tumor and adjacent non‐tumor regions, based on co‐registered H&E images, which were normalized to eliminate variations caused by uneven tissue thickness and heterogeneous component distribution.

### Statistical Analysis

The statistical methods used for each analysis and sample sizes are described in the figure legends. The statistical significance of comparisons between the two groups of data was assessed using an unpaired two‐tailed Student's *t*‐test. The statistical significance of comparisons between multiple groups was determined by two‐way ANOVA combined with Tukey's multiple comparison using GraphPad Prism (version 10.0). Differences were considered statistically significant if the *p*‐value was less than 0.05. Significance was indicated as: *p < 0.05, **p < 0.01, ***p < 0.001, ****p < 0.0001; ns, not significant. All statistical results were presented as the mean ± S.E.M. All experiments were repeated at least three times, yielding consistent results.

## Conflict of Interest

The authors declare no conflict of interest.

## Author Contributions

Y.M. and J.‐J.Z. contribute equally to this study. W.X. and S.M.Z. conceived the project and wrote the manuscript. Y.Z.M., J.J.Z., M.Q.Y., H.W., Y.F., H.L.Z., Y.Y.Q., Q.Z., M.W., G.L., Z.F.G., and M.X. performed the biochemical cell biologic experiments. Y.L. performed MS analysis. P.C.L., J.L., Y.Y., and Y.K. supervised all experiments. All authors contributed to the article and approved the submitted version.

## Supporting information



Supporting Information

Supporting Information

Supporting Information

Supporting Information

Supporting Information

Supporting Information

## Data Availability

The data that support the findings of this study are available from the corresponding author upon reasonable request.
