# Supplementary material for: CDH1 Orchestrates Anabolic Events to Promote Cell Cycle Initiation
Source: Adv Sci (Weinh). 2025 Sep 29;12(47):e07584. doi: 10.1002/advs.202507584 (PMC12713023; doi:10.1002/advs.202507584)
Supplement: Supplementary file 1 — Supporting Information [file ADVS-12-e07584-s003.docx]

**C**

**D**

*De novo*

*purine synthesis*

PRPP

IMP

AMP

XMP

ADP

dADP

dATP

dGTP

dGDP

GDP

GMP

IMPDH

GMPS

Mizoribine

*De novo pyrimidine synthesis*

dCTP

dUTP

dTMP

dTTP

dUMP

UMP

Orotate

Dihydroorotate

Glutamine

DHODH

Teriflunomide

Orotidine-5-P

PRPP

R5P

Ki67

Actin

Conflu(%)

50

100

HeLa


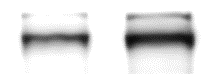

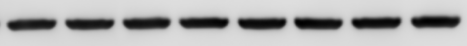


kDa

-300

-40

Confluent

Exponential

HeLa

HeLa

HeLa

**E**

**I**

**K**

**F**

HeLa

HeLa

HeLa

**G**

**H**

**J**

Ect1/E6E7

**A**


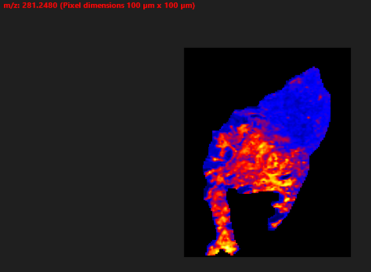


FA 18:1


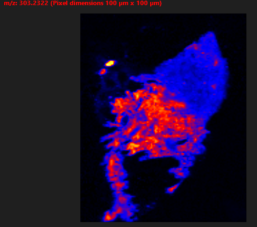


FA 20:4


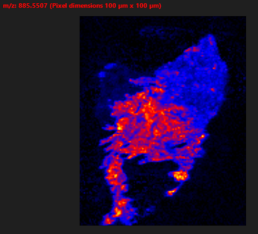


PI 38:4


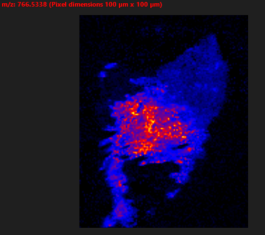


PE 38:4

**B**

SiHa

**Figure S1.** Blocking nucleotides synthesis inhibits cell proliferation. A) MSI revealed increased fatty acids in cervical cancer tissue. Fatty acids (FA18:1, FA20:4, PI38:4, and PE38:4) in both cancer and adjacent non-cancer tissues were detected using MSI. Scale bar, 2 mm. The quantification of the images was shown in Figure S1B, Supporting Information. B) Quantification of metabolites listed in Figure 1A and Figure S1A, Supporting Information (n=2). C-D) Targeted metabolites assay revealed decreased oxidative PPP metabolites and increased nucleotides in proliferating cells. The levels of oxidative PPP metabolites, non-oxidative PPP, nucleotides, and glycolytic metabolites were compared using targeted LC-MS between exponential growing and confluent grown SiHa cells (C) and Ect1/E6E7 cells (D) (n=3). E) Schematic of sampling exponentially growing HeLa cells and confluent HeLa cells (n=6). Ki67 was used as the proliferation marker. F-H) PPP flux is increased in proliferating cells. The activities of G6PD (F) and TKT (G), and the levels of NADPH (H) were compared between exponential growing and confluent grown HeLa cells (n=3). I) Targets of mizoribine and teriflunomide in nucleotide synthesis are illustrated. J) Mizoribine and teriflunomide co-treatment decreased nucleotide synthesis levels. Nucleotide levels were measured after mizoribine and teriflunomide for 6 hrs (n=3). K) Blocking nucleotide synthesis decreased cell proliferation. HeLa cell growth was measured using a CCK-8 assay kit in the absence and presence of mizoribine and teriflunomide (n=6). All data are presented as mean ± S.E.M. Statistical significance was assessed using two-tailed paired (B) and unpaired Student’s t-test (C,D,F,G,H,J,K). **p* < 0.05, ***p* < 0.01, ****p* < 0.001, *****p* < 0.0001; ns, not signficant.

**A**


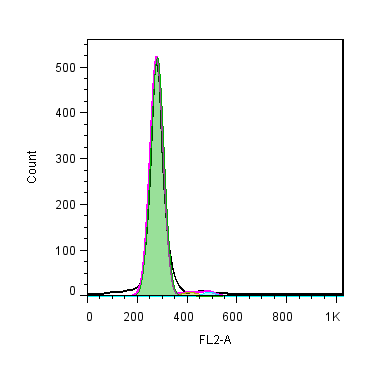

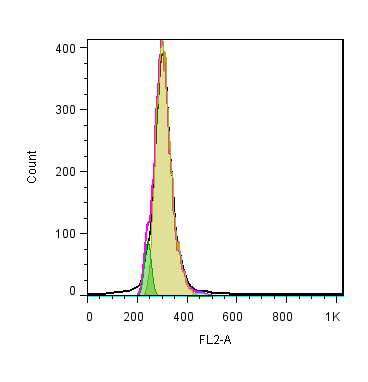

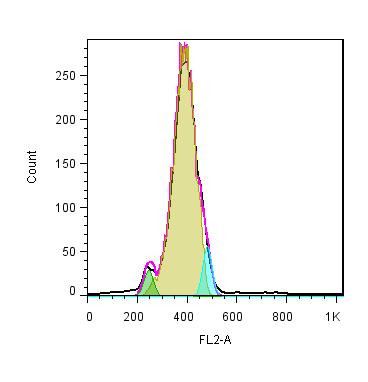

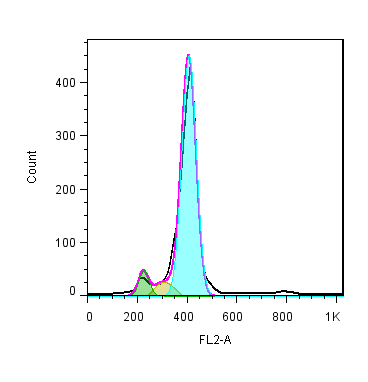

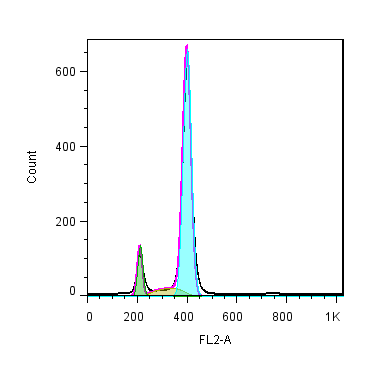

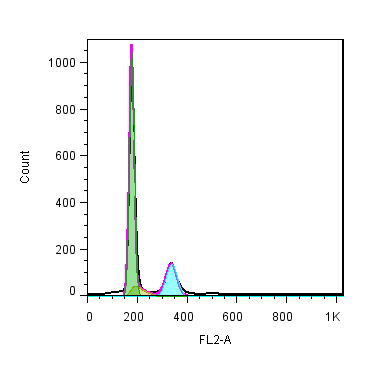

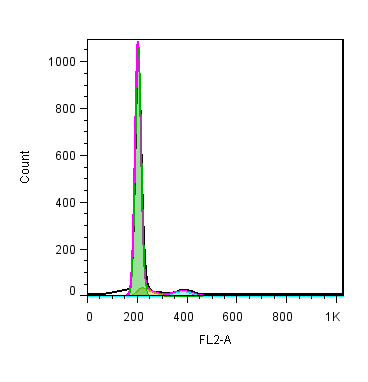

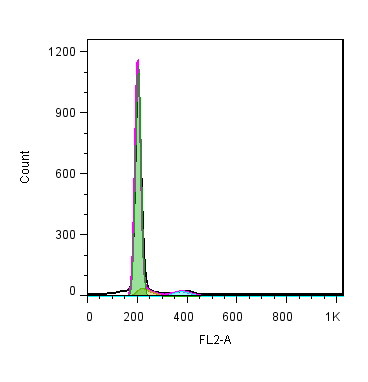


FL2-A(10^2^)

0

2

4

6

8

0

100

200

300

400

500

Counts

0

2

4

6

8

10

12

14

Time after DTB release (hrs)

0

2

4

6

8

0

2

4

6

8

0

2

4

6

8

0

2

4

6

8

0

2

4

6

8

0

2

4

6

8

0

2

4

6

8

**F**

HeLa

HeLa

**B**

**C**

**D**

**E**

100

50

Conflu(%)

SiHa

CDH1

VHL

HIF1*α*

AARS2

TKTL1

Actin

kDa

-50

-25

-70

-100

-100

-40


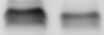

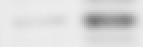

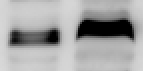

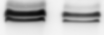

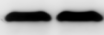

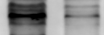


Actin

Flag

kDa

-50

-40

SiHa


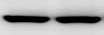

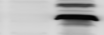

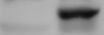


Ect1/E6E7

kDa

-50

-40

Actin

Flag


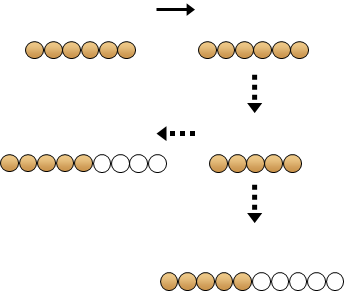


**^13^C_6_-Glucose**

**G-6-P**

**R-5-P**

**UMP**

**IMP**

**G**

**H**

**I**

**J**

100

50

Conflu(%)

CDH1

VHL

HIF1*α*

AARS2

TKTL1

Actin

Ect1/E6E7


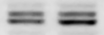

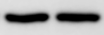

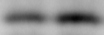

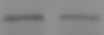

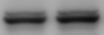

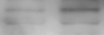


kDa

-50

-25

-70

-100

-100

-40


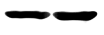


*TKTL1* KO HeLa

R5P

(M+5)

IMP

(M+5)

UMP

(M+5)

*CDH1* KD HeLa

R5P

(M+5)

IMP

(M+5)

UMP

(M+5)

*VHL* KO HeLa

R5P

(M+5)

IMP

(M+5)

UMP

(M+5)

**Figure S2.** TKTL1-CDH1-VHL axis regulates R5P. A) DTB-synchronized HeLa cells (time 0) were released by removing thymidine, and the fractions of cells in different cell cycle phases were analyzed using flow cytometry (n=3). B-C) CDH1 increases R5P levels. The levels of R5P were measured using LC-MS in SiHa (B) and Ect1/E6E7 cells (C) with or without CDH1 overexpression (n=3). D-E) High CDH1 levels in proliferating cells. Levels of CDH1, VHL, TKTL1, HIF1*α* and AARS2 were detected in exponentially and confluently grown SiHa (D) and Ect1/E6E7 cells (E). F) Media R5P supplementation increased cellular R5P level. R5P levels were measured in HeLa cells cultured with or without 10 mM R5P supplementation (n=3). G) Schematic of the conversion of ^13^C-glucose to ^13^C-R5P, ^13^C-IMP and ^13^C-UMP. H-J) The role of TKTL1-CDH1-VHL axis in nucleotide biosynthesis. The levels of ^13^C-labeled R5P, IMP and UMP were measured using LC-MS in exponentially and confluently grow *TKTL1* KO (H), *CDH1* knockdown (I) and *VHL* KO HeLa cells (J) (n=3). Statistical significance was assessed using two-tailed unpaired Student’s t-test (B,C,F,H,I,J) . **p* < 0.05, ***p* < 0.01, ****p* < 0.001; ns, not significant.

**C**

**A**

**E**

**D**


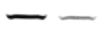


+

−

si*G6PD*

HeLa

CDH1

VHL

TKTL1

Actin


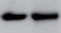

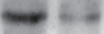

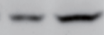


G6PD


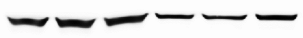


kDa

-50

-25

-70

-50

-40

UB-HA

VHL-Flag

R5P

MG132

IP: Flag

Input

HA

Flag

Flag

Actin


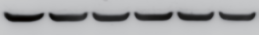

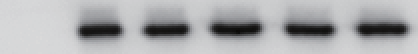

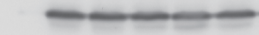

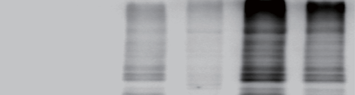


−

+

+

+

+

+

+

−

+

+

+

+

−

−

+

−

+

−

HeLa

−

−

−

−

+

+

kDa

-100

-70

-50

-25

-40

-25

-25

-40

UB-HA

VHL-Flag

−

+

+

+

+

+

−

+

+

+

−

−

+

−

−

HeLa

si*G6PD*

MG132

−

−

−

−

+

IP: Flag

Input

HA

Flag

Flag

Actin

G6PD


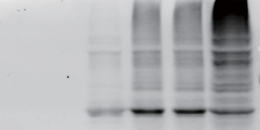

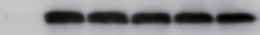

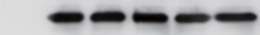

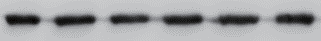

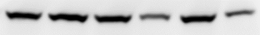


+

+

+

+

kDa

-100

-70

-50

-25

-40

-25

-25

-40

-50

G6PD

Actin


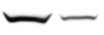

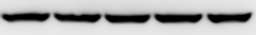


kDa

-50

-40

HeLa

HeLa

Actin

CDH1


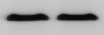

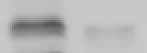


si*Scr.*

si*CDH1*

kDa

-50

-40

**F**


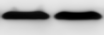

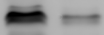

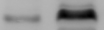


+

−

R5P

SiHa

CDH1

VHL

TKTL1

Actin

Cyclin A2

SKP2

CDC20

kDa

-50

-25

-70

-50

-50

-50

-40


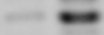

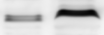

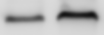

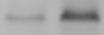


**B**

**Figure S3.** R5P decreases VHL ubiquitination. A) R5P decreases CDH1 levels. The cellular protein levels of CDH1 and its substrates VHL, TKTL1, Cyclin A2, SKP2, and CDC20 were measured in SiHa cells with or without R5P treatment. B) *G6PD* knockdown increases CDH1 and decreases CDH1 substrate levels. Levels of CDH1 and CDH1 substrates were measured in HeLa and *G6PD*-silencing HeLa cells. C) G6PD knockdown decreases R5P levels. R5P levels were determined in HeLa and *G6PD*-silencing HeLa cells (n=3). D-E) R5P and G6PD regulate VHL ubiquitination. VHL ubiquitination levels were determined in R5P-treated (C) or *G6PD*-silencing HeLa cells (D). F) *CDH1* knockdown speeds up the G1/S transition. The numbers of cells in the G1, S, and G2/M phases of HeLa cells were detected in HeLa and *CDH1*-silencing HeLa cells (n=3). All data are presented as mean ± S.E.M. Statistical significance was assessed using two-tailed unpaired Student’s t-test (C). ***p* < 0.01.

**A**

**B**

Req

0

50

100

150

200

250

0.008

0.004

Conc.(μM)

0

Req

Conc.(μM)

400

600

800

1000

200

0

**C**

**D**

*β*-TRCP1 degron motif:

(569)

(563)

TKTL1 sequence:

**D**

**S**

**G**

**X**

**X**

**X**

**S**

**V**

**S**

**G**

**V**

**P**

**Q**

**S**

*β*-TRCP1-Myc

+

−

+

−

+

+

CDH1-Flag

R5P

−

−

+

+

+

+

−

−

−

−

−

+

−

−

−

−

+

−

TKTL1-HA

TKTL1^MUT^-HA

−

−

−

−

−

−

+

+

+

+

+

−

+

−

−

+

*In vitro* pull down

+

+

+

−

+

Flag

Myc

HA

Myc

Flag


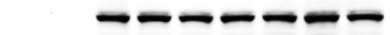

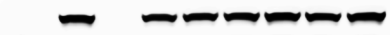

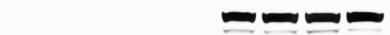

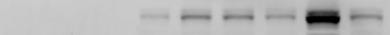

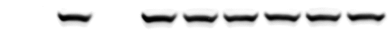


IP: Flag

Input

kDa

-70

-50

-70

-70

-70


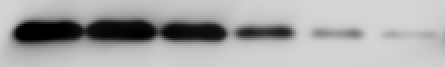

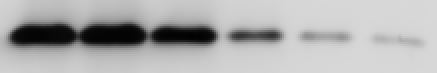

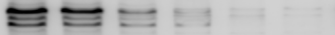

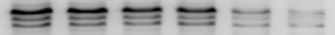


GAPDH

TKTL1

GAPDH

TKTL1

HeLa

NC

+R5P

46

52

58

64

70

76

Tem. (℃)


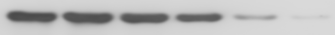

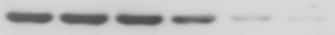

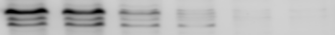

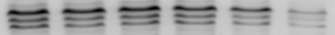


GAPDH

TKTL1

GAPDH

TKTL1

HeLa Cell Lysate

NC

+R5P

46

52

58

64

70

76

Tem. (℃)

CDH1 degron motif:

(26)

(18)

TKTL1 sequence:

**R**

**X**

**X**

**L**

**X**

**X**

**X**

**X**

**D**

**N**

**E**

**/**

**/**

**R**

**G**

**T**

**L**

**Q**

**V**

**L**

**Q**

**D**

**E**


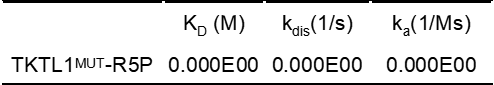

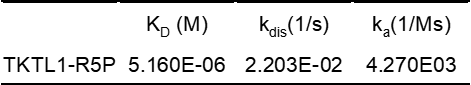


**Figure S4.** R5P binds to TKTL1 to enhance SCF*^β^*^-TRCP1^-mediated CDH1 degradation. A) R5P binds to TKTL1. Binding of R5P to recombinant TKTL1 was assayed using BLI. BLI, biolayer interferometry. B) Thermal stability of TKTL1 is increased by R5P. The thermal stability of TKTL1 was assayed in HeLa cell (left) or Hela cell lysate (right) treated with or without R5P. GAPDH was used as control. C) R5P doesn’t bind to TKTL1^MUT^. Binding of R5P to recombinant TKTL1^MUT^ was assayed using BLI. D) R5P promotes TKTL1-CDH1-*β*-TRCP1 interaction through binding to TKTL1. Interaction between recombinant CDH1 and *β*-TRCP1 was tested under the presence of either recombinant TKTL1 or TKTL1^MUT^ and the absence or presence of R5P in the solution. E) TKTL1 has recognizing motifs for both CDH1 and *β*-TRCP1. TKTL1 contains CDH1- (upper) and *β*-TRCP1- (lower) recognizing motifs. Consensus-recognizing residues are colored.

**C**

**H**


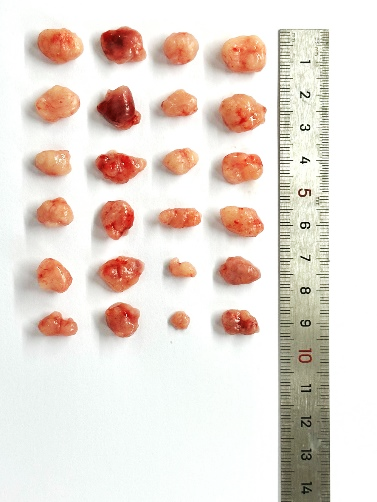

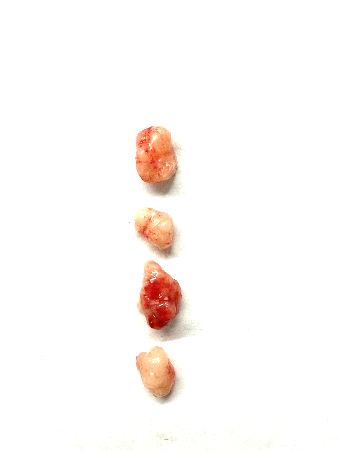


HeLa

*G6PD^-/-^*

R5P

injection

−

+

−

+


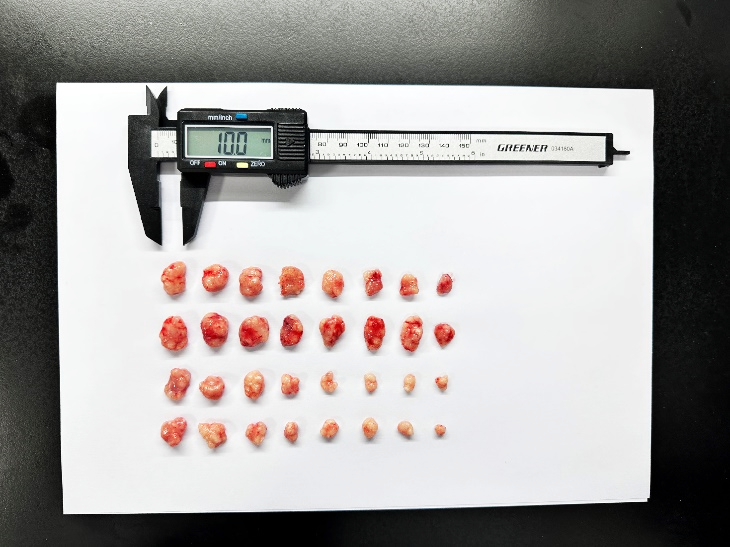


WT

G6PD^-/-^

sh*CDH1*

G6PD^-/-^

+sh*CDH1*

HeLa

**A**

sh*CDH1*

sh*Scram*

WT

*G6PD^-/-^*

HeLa Xenografts


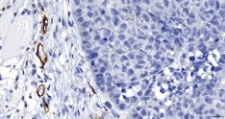

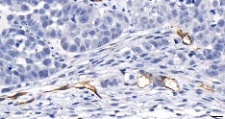

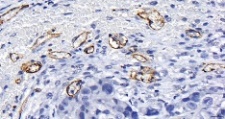

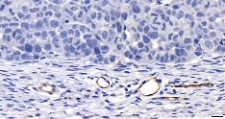


**L**

HeLa

BALB/c Nude Mice

CD31


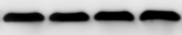


100

50

Conf(%)

SiHa

WT

si*CDH1*

100

50

HIF1*α*

CDH1

VEGF

Actin

kDa

-70

-40

-50

-40


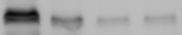

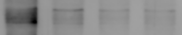

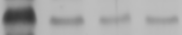


**B**

**G**


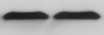


+

−

CDH1-Flag

SiHa

GLUT1

Flag

Actin

kDa

-50

-50

-40


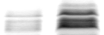

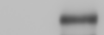


**M**

**N**

HeLa

**O**

+

−

*siG6PD*

HeLa

GLUT1

G6PD

Actin


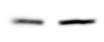

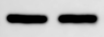

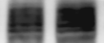


kDa

-50

-50

-40

**D**

**E**

**F**

**I**

**J**

**K**

**Figure S5.** R5P regulates angiogenesis via CDH1. A) High *VEGF* mRNA levels are not in *CDH1*-silenced proliferating cells. *VEGF* mRNA levels were measured in exponentially and stationarily growing HeLa and *CDH1*-silenced HeLa cells (n=4). B) High HIF1*α* and VEGF in proliferating CDH1-intact cells. Protein levels of HIF1*α* and VEGFA were detected in exponential growing and stationary stage SiHa and *CDH1*-silencing SiHa cells. C-F) R5P decreases tumor tissue angiogenesis. HeLa and *G6PD*^-/-^ HeLa cell BALB/c nude mouse xenografts were grown with or without R5P intraperitoneal injection. Vascularization was assessed by visualization (C). The tumor volume (D), food intake (E) and body weight (F) were recorded (n=6). G) R5P gavage increased mouse circulating R5P. R5P levels in the serum were detected after intraperitoneal injection of 3 mg/kg in BALB/c nude mice every other day for two weeks (n=4). H-I) R5P CDH1-dependently decreases angiogenesis. Surface blood vessels of HeLa, *G6PD*^-/-^ HeLa, and *G6PD*^-/-^/si*CDH1* HeLa BALB/c nude mouse xenografts were compared (H). The tumor volume (I), food intake (J) and body weight (K) were recorded (n=8). L) G6PD silencing CDH1-dependently increases angiogenesis. CD31 staining was employed to measure the levels of angiogenesis in HeLa, *G6PD*^-/-^ HeLa, and *G6PD*^-/-^/si*CDH1* HeLa cell BALB/c nude mouse xenografts (n=9). Scale bar, 50 μm. M) CDH1 increases GLUT1 expression. GLUT1 protein levels were detected in HeLa and CDH1-overexpressing SiHa cells. N) G6PD silencing increases GLUT1 expression. GLUT1 protein levels were detected in HeLa and *G6PD*-silenced HeLa cells. O) CDH1 upregulates GLUT1 transcriptional levels via HIF1*α*. The mRNA levels of *GLUT1* were measured in HeLa and *HIF1α* KO HeLa cells with or without CDH1 overexpression (n=3). All data are presented as mean ± S.E.M. Statistical significance was assessed using two-way ANOVA (A,D,E,F,I,J,K,O) and two-tailed unpaired Student’s t-test (G). **p* < 0.05, ***p* < 0.01, ****p* < 0.001, *****p* < 0.0001; ns, not significant.

**a**

**f**

**g**


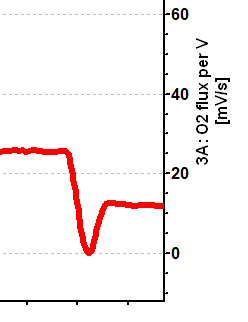

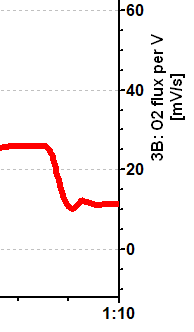

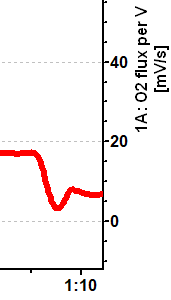

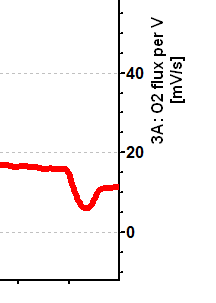


O_2_ flux per V [mV/S]

si*G6PD*

*AARS2* KO

−

−

+

−

+

+

−

+

HeLa

CDH1-Flag

*AARS2* KO

−

−

+

−

+

+

−

+


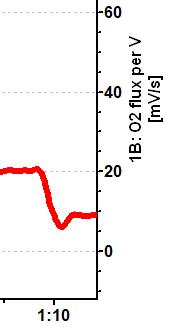

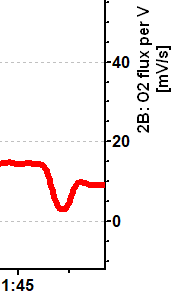

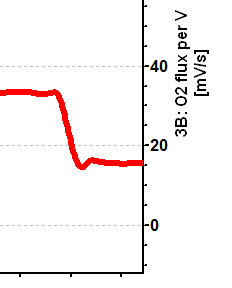

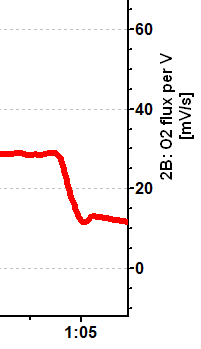


O_2_ flux per V [mV/S]

HeLa

**b**

**c**

**d**

**e**

**h**

+

−

+

CPT2-Flag

−

−

+

HeLa

Lac-K457/8

Flag

Flag

CDH1-Myc

Myc


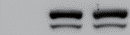

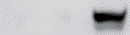

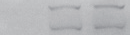

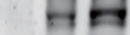


kDa

-70

-70

-70

-50

+

−

+

CPT2-Flag

−

−

+

HeLa

Lac-K457/8

Flag

Flag

si*CDH1*

CDH1

Actin

kDa

-70

-70

-70

-50

-40

+

−

+

CPT2-Flag

−

−

+

HeLa

Lac-K457/8

Flag

Flag

si*G6PD*

G6PD

Actin

kDa

-70

-70

-70

-50

-40

Flag

Flag

Myc

Lac-K457/8

+

−

+

CPT2-Flag

−

−

+

HeLa

VHL-Myc

kDa

-70

-70

-70

-25

si*G6PD*

+

−

HeLa

PDHA1

PDHA1-pS300

G6PD

Actin

PDHA1-pS293

PDHA1-pS232

kDa

-40

-40

-40

-40

-50

-40

**Figure S6.** R5P and CDH1 AARS2-dependently regulate OXPHOS. A) Schematic diagram showing that CDH1 downregulation inhibits OXPHOS by activating AARS2, which inhibits AcCoA influx from PDHA1 and CPT2. B-E) CDH1, VHL, and R5P all regulate CPT2 Lac-K457/8. Lac-K457/8 levels were compared for ectopically expressed CPT2 between HeLa and CDH1-overexpressing (B), *CDH1*-silencing (C), *G6PD*-silencing (D), and VHL-overexpressing (E) HeLa cells. F) R5P has no effect on PDHA1 phosphorylation. Ser232, Ser293, and Ser300 phosphorylation of PDHA1 was determined in HeLa and *G6PD*-silencing HeLa cells. G-H) R5P and CDH1 AARS2-dependently regulate cell oxygen consumption. Impact of *G6PD*-silencing (G) and CDH1 overexpression (H) on the OCRs was measured in both HeLa and *AARS2* KO HeLa cells.

**A**

**E**

HeLa

Control

R5S

P

T

P

T

P

T

P

T

P

T

P

T

Mice Liver Cancer Tissue

P

T

P

T

P

T

P

T

Cleaved caspase-3

Mice liver cancer tissue

HeLa

+

−

R5S

R5P

−

−

+

−

+

+

**B**

**C**

**D**

**F**

**G**

**Figure S7.** R5S sensitizes chemotherapy. A) R5P rescues R5S-induced DNA damage. The representative image of Comet assay results related to Figure 7H. Scale bar, 100 μm. B-C) R5S induces more pronounced apoptosis in liver cancer tissue. Levels of cleaved caspase-3 were compared between mouse liver cancer tissue and its adjacent non-cancer tissue. The quantification (B) and more images (C) related to Figure 7K (n=5). Scale bar, 50 μm. D) R5S sensitizes 5-FU-induced apoptosis more potently in proliferating HeLa cells. Effects of 5-FU on the apoptotic rates were measured in proliferating and confluent HeLa cells with or without R5S treatment (n=3). E) R5S sensitizes cisplatin to inhibit xenograft growth. Volumes of untreated, R5S- or cisplatin-treated, and R5S- and cisplatin-treated HeLa xenografts were measured at the indicated time points (n=6). F-G) Food intake (F) and body weight (G) were recorded in mice related to Figure 7M. All data are presented as mean ± S.E.M. Statistical significance was assessed using two-tailed unpaired Student’s t-test (B) and two-way ANOVA (D,E,F,G). **p* < 0.05, ***p* < 0.01, ****p* < 0.001, *****p* < 0.0001.
